# Hepatic glycogen directly regulates gluconeogenesis through an AMPK/CRTC2 axis in mice

**DOI:** 10.1172/JCI188363

**Published:** 2025-06-02

**Authors:** Bichen Zhang, Morgan M. Johnson, Timothy Yuan, Tammy-Nhu Nguyen, Junichi Okada, Fajun Yang, Alus M. Xiaoli, Liana H. Melikian, Songran Xu, Benyamin Dadpey, Jeffrey E. Pessin, Alan R. Saltiel

**Affiliations:** 1 Department of Medicine, University of California, San Diego, San Diego, California, USA.; 2 Department of Medicine and; 3 Department of Molecular Pharmacology, Fleischer Institute for Diabetes and Metabolism, Albert Einstein College of Medicine, Bronx, New York, USA.; 4 Department of Pharmacology, University of California, San Diego, San Diego, California, USA.

**Keywords:** Cell biology, Metabolism, Gluconeogenesis, Glucose metabolism, Signal transduction

## Abstract

Glycogenolysis and gluconeogenesis ensure sufficient hepatic glucose production during energy shortages. Here, we report that hepatic glycogen levels control the phosphorylation of a transcriptional coactivator to determine the amplitude of gluconeogenesis. Decreased liver glycogen during fasting promotes gluconeogenic gene expression, while feeding-induced glycogen accumulation suppresses it. Liver-specific deletion of the glycogen scaffolding protein, protein targeting to glycogen (PTG), reduces glycogen levels, increases the expression of gluconeogenic genes, and promotes glucose production in primary hepatocytes. In contrast, liver glycogen phosphorylase (PYGL) knockdown or inhibition increases glycogen levels and represses gluconeogenic gene expression. These changes in hepatic glycogen levels are sensed by AMP-activated protein kinase (AMPK). AMPK activity is increased when glycogen levels decline, resulting in the phosphorylation and stabilization of CREB-regulated transcriptional coactivator 2 (CRTC2), which is crucial for the full activation of the cAMP-responsive transcriptional factor CREB. High glycogen allosterically inhibits AMPK, leading to CRTC2 degradation and reduced CREB transcriptional activity. Hepatocytes with low glycogen levels or high AMPK activity show higher CRTC2 protein levels, priming the cell for gluconeogenesis through transcriptional regulation. Thus, glycogen plays a regulatory role in controlling hepatic glucose metabolism through the glycogen/AMPK/CRTC2 signaling axis, safeguarding efficient glucose output during fasting and suppressing it during feeding.

## Abstract 2

Liver glycogen levels regulate glucose output by controlling a signaling pathway involving AMPK and CRTC2, ensuring sufficient glucose production during fasting and feeding.

## Introduction

The precise regulation of plasma glucose results from a balance between glucose absorption in the intestine, production by the liver and kidneys, and uptake and metabolism in peripheral tissues. The liver is responsible for 90% of endogenous glucose production, resulting from glycogenolysis and gluconeogenesis, which are thought to act in parallel to provide sufficient hepatic glucose output during fasting (1). Numerous studies have concentrated on elucidating the underlying mechanisms of gluconeogenesis, focusing on the hormonal control of substrate flux and gluconeogenic gene transcription. Although glycogen is the first choice for energy storage and mobilization in the liver, our understanding of the dynamics and impact of glycogen metabolism is far from complete.

Protein targeting to glycogen (PTG) is a member of a family of critical scaffolding proteins that control glycogen metabolism. The enzymes glycogen synthase (GS) and glycogen phosphorylase (PYGL) that catalyze glycogen synthesis and breakdown assemble in a signaling complex with PTG. Upon feeding, protein phosphatase 1 (PP1) is directed to the complex, facilitating the dephosphorylation of GS and PYGL to increase net glycogen accumulation (2, 3). Overexpression of PTG increases PP1 activity, which leads to dephosphorylation of GS and PYGL, and increases glycogen synthesis and blocks breakdown, since PYGL bound to PTG is resistant to glycogenolytic agents like glucagon or cyclic AMP (cAMP) (4). Importantly, analysis of multiple large-scale human GWAS and whole-exome sequencing studies shows that *PPP1R3C*, the gene encoding PTG, is strongly associated with fasting glucose after adjustment for BMI (5). Moreover, hepatic glycogen was recently reported to be lower in individuals with type 2 diabetes, which is associated with their increased postprandial glucose production (6).

In addition to regulation of glycogenolysis, hepatic glucose metabolism is governed by a highly organized pathway for gluconeogenesis consisting of multiple layers of regulation. While gluconeogenesis is acutely controlled by substrate availability during fasting via increased lipolysis in adipose tissue, longer-term regulation involves changes in gluconeogenic gene expression. These genes are controlled by cAMP-responsive element–binding protein (CREB), the activity of which is increased by protein kinase A–mediated phosphorylation (7). CREB functions in concert with a family of latent cytoplasmic coactivators called CREB-regulated transcriptional coactivators (CRTCs) that are also regulated by phosphorylation (8, 9). In the liver, CRTC2 holds a unique position in this process, shuttling between the cytoplasm and nucleus under feeding and fasting conditions. Under fed conditions, CRTC2 is phosphorylated on Ser^171^ and Ser^307^ by salt-inducible kinase 2 (SIK2) and sequestered in the cytoplasm. In fasting, SIK2 activity is inhibited by cAMP, and thus glucagon produces reduced phosphorylation of the SIK2 sites on CRTC2, permitting its entrance to the nucleus, where it binds to CREB (10). CRTC2 serves as a crucial coactivator for CREB, promoting the transcription of a group of gluconeogenic genes that contain a CRE-binding motif. It has been shown that CRTC2 expression correlates with energy status, and interestingly, the abundance of CRTC2 determines the amplitude of the gluconeogenic response (11, 12). However, whether CRTC2 synthesis or stability is under hormonal or nutritional control has not been investigated.

Here, we report that hepatic glycogen levels tune the gluconeogenesis pathway through an AMP-activated protein kinase (AMPK)/CRTC2 signaling axis, providing a safeguard for appropriately controlling gluconeogenesis. Depletion of hepatic glycogen by targeted deletion of PTG stimulates gluconeogenesis in a cell-autonomous fashion, whereas accumulation of glycogen suppresses gluconeogenesis. The feeding- and fasting-induced changes in cellular glycogen levels are sensed by AMPK. Low glycogen levels result in increased activity of AMPK, which phosphorylates CRTC2 at Ser^349^, increasing CRTC2 protein stability and expression, thus amplifying gluconeogenic gene expression. Together, these data reveal a physiological mechanism by which the liver coordinates both glycogen and gluconeogenesis pathways to adjust glucose metabolism economically. Thus, glycogen serves a regulatory function, ensuring that high glycogen levels repress gluconeogenic gene expression during conditions of excess energy, while glycogen depletion permits the induction of these genes to restore glucose homeostasis.

## Results

### Hepatic glycogen levels regulate gluconeogenesis in a cell-autonomous fashion.

**Figure 1:**
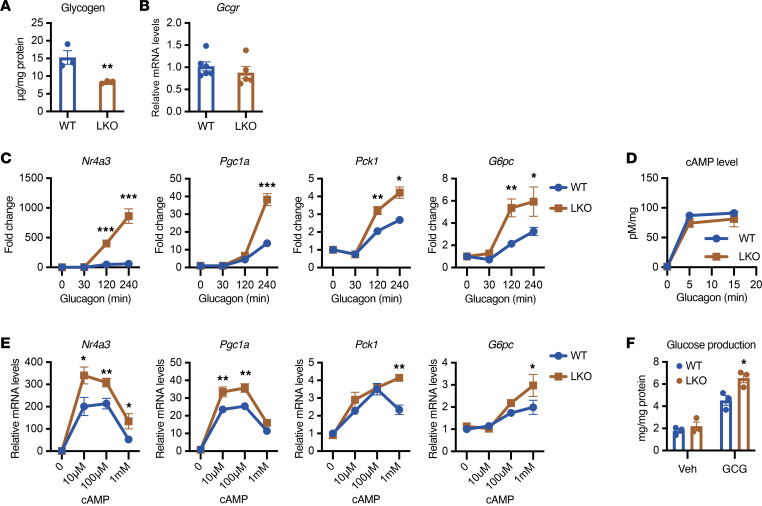
Depletion of hepatocellular glycogen induces gluconeogenic expression and glucose production. (**A**) Glycogen levels in WT and PTGLKO primary hepatocytes. (**B**) Glucagon receptor (*Gcgr*) gene expression in WT and PTGLKO hepatocytes. (**C**) Gene expression in WT and PTGLKO primary hepatocytes treated with 100 nM glucagon for indicated times. (**D**) cAMP levels in WT and PTGLKO primary hepatocytes. (**E**) Gene expression in WT and PTGLKO primary hepatocytes treated with different doses of cell-permeable cAMP. (**F**) Glucagon-stimulated glucose production in WT and PTGLKO hepatocytes. *n* = 3–6 per group. *P < 0.05; **P < 0.01; ***P < 0.001 by unpaired Student’s *t* test.

PTG is a molecular scaffold that directly binds to GS and PYGL, and the phosphatases and kinases that regulate their activity, assembling a signaling unit that is compartmentalized to the site of glycogen accumulation (2, 3, 13). In addition to controlling glycogen synthesis and breakdown, nutritional regulation of PTG gene expression also affects glycogen levels; *Ppp1r3c* expression in the liver is reduced during fasting and increased by feeding, and dramatically enhanced by high-fat diet feeding (14, 15). To investigate how glycogen levels affect hepatic glucose metabolism, we crossed PTG-floxed mice with *Albumin-Cre* mice to create mice with liver-specific knockout of PTG (PTGLKO) (Supplemental Figure 1, A–C; supplemental material available online with this article; https://doi.org/10.1172/JCI188363DS1). Deletion of PTG led to a 50% reduction of glycogen levels in primary hepatocytes isolated from PTGLKO mice compared with their WT littermates with no change observed in glucagon receptor expression (Figure 1, A and B, and Supplemental Figure 1D). We examined the expression of gluconeogenic genes and their regulators in WT and PTGLKO primary hepatocytes in response to glucagon. Expression of the gluconeogenic genes *Pck1* and *G6pc* is controlled by the activities of *Nr4a3* and *Pgc1a* (16, 17). Glucagon produced a 2-fold increase in expression of *Pck1 and G6pc* after 4 hours, and surprisingly, this effect was doubled in PTGLKO mice, with no effect on basal levels (Figure 1C). Interestingly, there was higher induction by glucagon of the upstream regulators of *Pck1* and *G6pc*, *Nr4a3* and *Pgc1a*, in PTGLKO hepatocytes, indicating that PTG plays a role in controlling gluconeogenic gene expression (Figure 1C).

Glucagon action is initiated by binding to its receptor, leading to the rapid activation of adenylyl cyclase, generating the second messenger cAMP, which in turn activates protein kinase A (PKA) that catalyzes phosphorylation of CREB to drive gluconeogenic gene expression (18). To understand how PTG knockout enhances this signaling cascade, we first examined the upstream signaling events. Examination of glucagon-stimulated cAMP levels in isolated primary hepatocytes revealed no differences between WT and PTGLKO hepatocytes, suggesting that PTG affects gluconeogenesis downstream of cAMP (Figure 1D). Treatment of primary hepatocytes with different doses of a cell-permeable cAMP analog (8-Br-cAMP) also revealed significantly higher gluconeogenic gene expression in PTGLKO compared with WT primary hepatocytes (Figure 1E). We also measured cellular glucose production by supplying fasted primary hepatocytes with lactate, pyruvate, and glutamine with treatment by glucagon. Glucagon-stimulated glucose production was increased in PTGLKO compared with WT hepatocytes with no difference observed in the basal level (Figure 1F).

**Figure 2:**
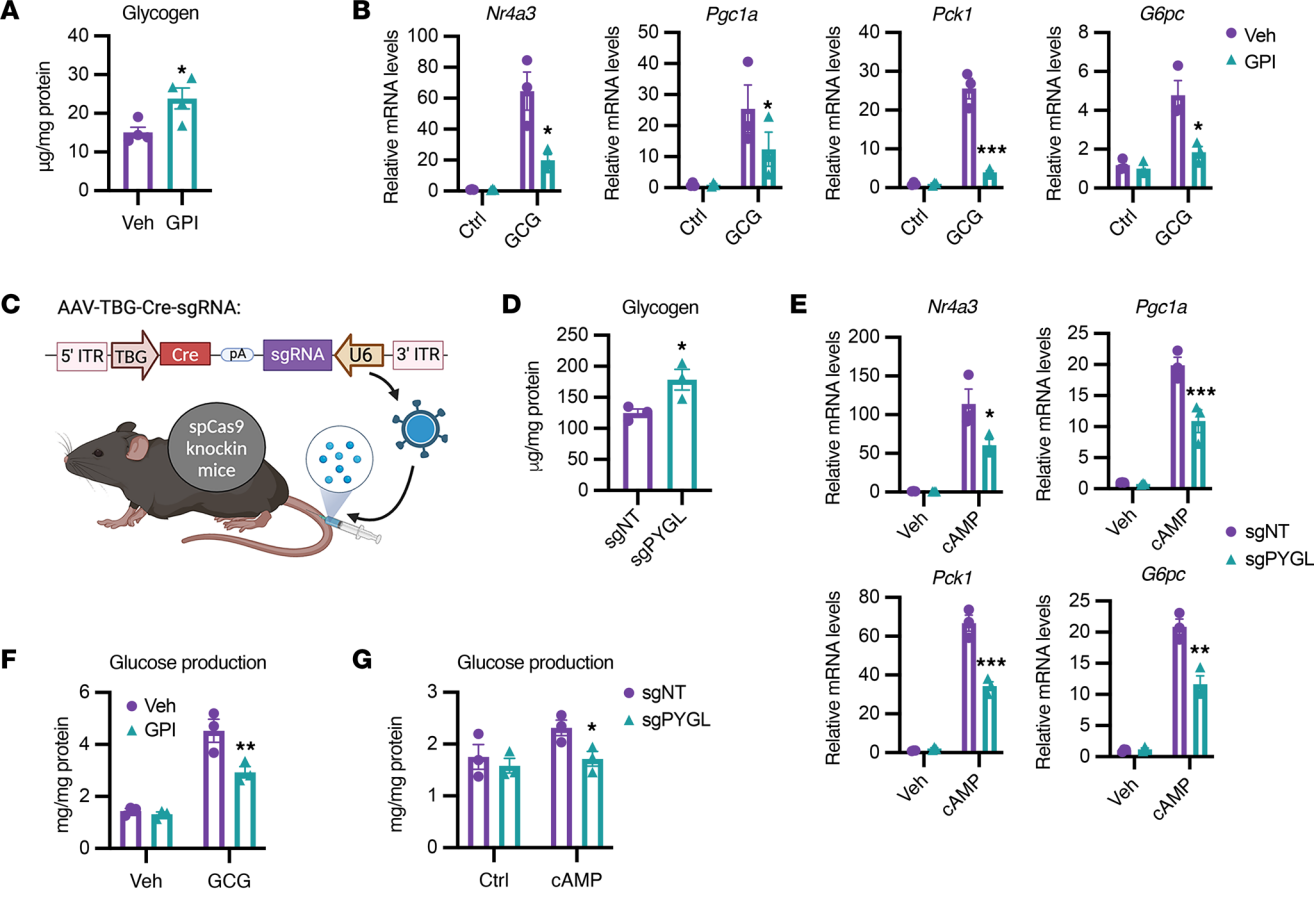
Hepatic glycogen levels regulate gluconeogenesis in a cell-autonomous fashion. (**A**) Glycogen levels in WT hepatocytes treated with vehicle (Veh) or glycogen phosphorylase inhibitor (GPI). (**B**) Gluconeogenic gene expression in vehicle- and GPI-pretreated hepatocytes. Cells were treated with vehicle or GPI overnight and then treated with or without 100 nM glucagon for 4 hours. (**C**) Schematic model of CRISPR-mediated glycogen phosphorylase (PYGL) knockdown in mice. (**D**) Glycogen levels in hepatocytes isolated from mice injected with single guide RNA targeting PYGL (sgPYGL) or non-targeting (sgNT) controls. (**E**) Gluconeogenic gene expression in primary hepatocytes isolated from sgNT and sgPYGL mice treated with or without 1 mM cAMP for 4 hours. (**F** and **G**) Glucagon-stimulated glucose production in vehicle- and GPI-treated hepatocytes (**F**); and cAMP-induced glucose production in sgNT and sgPYGL hepatocytes (**G**). *n* = 3–4 per group. *P < 0.05; **P < 0.01; ***P < 0.001 by unpaired Student’s *t* test. Panel **C** was created in BioRender (https://BioRender.com/x39s385).

To understand whether the effects of PTG deletion on gluconeogenic gene expression can be ascribed to glycogen levels, we used both genetic and pharmacological tools to increase cellular glycogen levels and examined whether accumulation of glycogen suppressed gluconeogenesis in a cell-autonomous fashion. We first used a specific glycogen phosphorylase inhibitor (GPI), which increased hepatocellular glycogen levels by about 30% (Figure 2A) (19). Pretreatment of hepatocytes with GPI suppressed the induction of gluconeogenic genes in response to glucagon (Figure 2B). GPI treatment did not affect the expression of glucagon receptor or cellular cAMP levels (Supplemental Figure 1, D–F). We also used adeno-associated virus–mediated (AAV-mediated) in vivo gene editing to inactivate glycogen phosphorylase (*Pygl*) specifically in hepatocytes by injecting AAV8 containing TBG-Cre–driven single guide RNA (sgRNA) targeting *Pygl* into spCas9 knockin mice flanked by the loxP-STOP-loxP site (Figure 2C) (20). We isolated primary hepatocytes from spCas9 mice injected with AAV8-TBG-Cre-sgPYGL (sgPYGL) and control mice injected with non-targeting sgRNA (sgNT). The knockdown of Pygl was confirmed by Western blot assay (Supplemental Figure 1G), and glycogen levels were significantly higher in sgPYGL hepatocytes compared with sgNT hepatocytes (Figure 2D). Similarly to GPI-treated hepatocytes, sgPYGL hepatocytes showed lower expression of gluconeogenic genes (*Nr4a3*, *Pgc1a*, *Pck1*, and *G6pc*) compared with sgNT hepatocytes in response to glucagon (Figure 2E). Moreover, glucagon-stimulated glucose production was blunted in both GPI-treated cells and sgPYGL hepatocytes in comparison with their respective controls (Figure 2, F and G). These results demonstrate that glycogen depletion sensitizes hepatocytes to catabolic signals, producing the amplification of gluconeogenic gene expression and glucose output, whereas accumulation of glycogen suppresses gluconeogenesis; these effects of glycogen occur downstream of cAMP signaling in a cell-autonomous manner.

### Gluconeogenic gene expression is regulated by glycogen levels in vivo.

**Figure 3:**
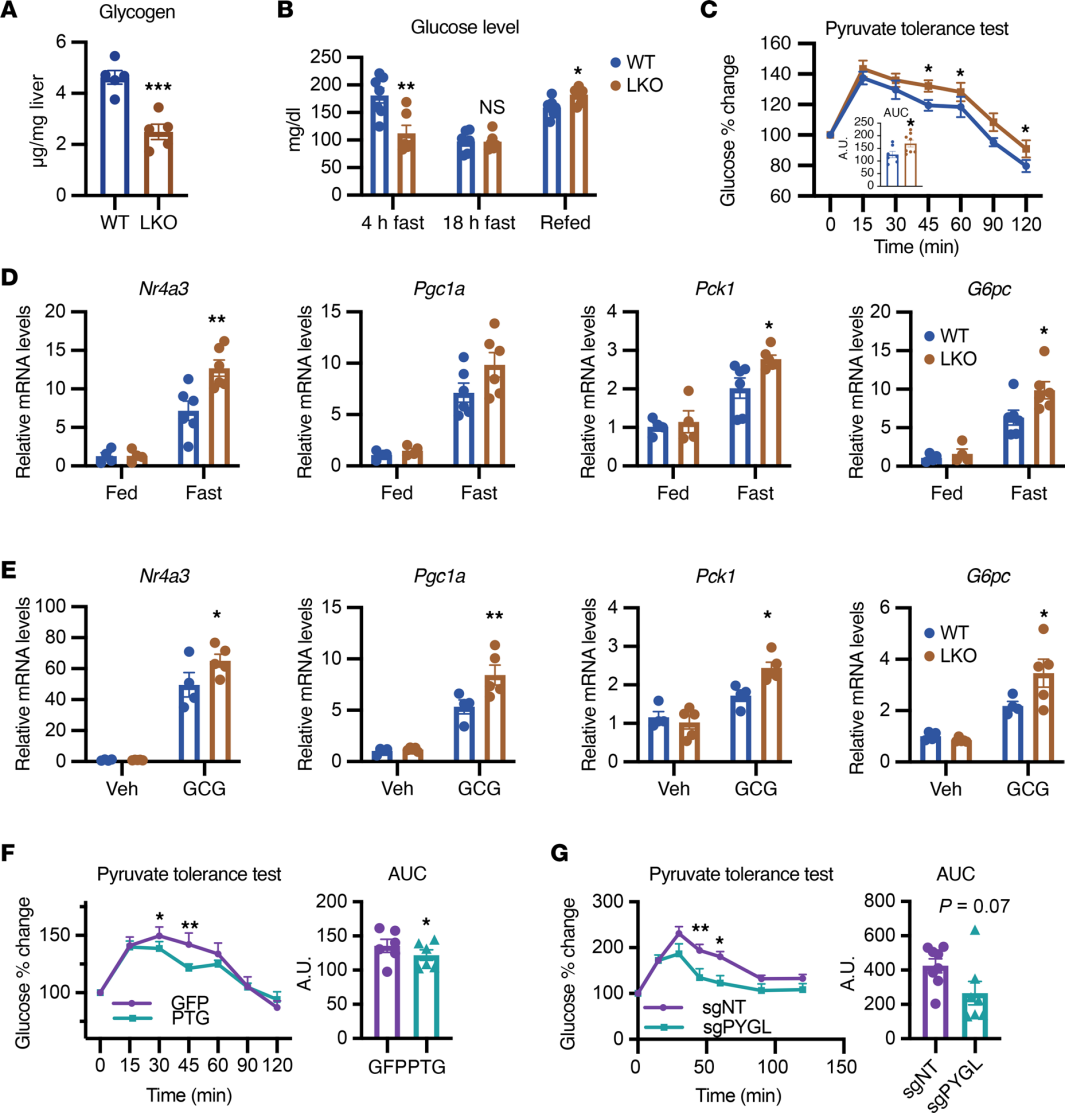
Glucose metabolism is regulated by glycogen levels in vivo. (**A**) Glycogen levels in liver lysates from WT and PTGLKO mice. Mice were fasted 4 hours before sacrifice. (**B**) Glucose levels in WT and PTGLKO mice. (**C**) Pyruvate tolerance test (PTT) and quantification of area under the curve (AUC). Mice were injected with 1.5 g sodium pyruvate per kilogram body weight. (**D**) Gluconeogenic gene expression in fasted and fed WT and PTGLKO mice. (**E**) Gluconeogenic gene expression in liver lysates from WT and PTGLKO mice. Mice were fasted for 1 hour, injected with 0.5 mg/kg glucagon, and sacrificed 30 minutes after. (**F**) PTT assay of mice injected with AAV-GFP and AAV-PTG, and quantification of AUC. (**G**) PTT assay of sgNT and sgPYGL mice and quantification of AUC. *n* = 3–8 per group. *P < 0.05; **P < 0.01; ***P < 0.001 by unpaired Student’s *t* test (**A**–**C** and **E**–**G**) and 2-way ANOVA (**D**).

PTGLKO mice showed normal body weight, body composition, energy expenditure, and food intake compared with WT littermates in the fed state (Supplemental Figure 2, A–D). Liver lysates from PTGLKO mice showed decreased glycogen levels, confirmed by periodic acid–Schiff staining (Figure 3A and Supplemental Figure 2E). Serum glucose levels in WT and PTGLKO mice showed different patterns depending on their nutritional state. Glucose levels are maintained by hepatic glycogenolysis during short-term fasting. PTGLKO mice showed lower glucose levels after 4 hours of fasting compared with WT mice, likely a reflection of reduced hepatic glycogen storage during feeding (Figure 3B). In contrast, PTGLKO and WT mice showed similar glucose levels after overnight fasting, indicating that increased gluconeogenesis in PTGLKO mice compensated for the lower glycogenolysis to achieve sufficient glucose production for euglycemia (Figure 3B). Interestingly, circulating amino acid levels were lower in PTGLKO mice after overnight fasting, consistent with the role of amino acids as a substrate for gluconeogenesis (Supplemental Figure 2F) (21, 22). In contrast, serum glycerol levels were comparable between WT and PTGLKO mice (Supplemental Figure 2G). While other compensatory mechanisms may be involved in the regulation of glucose levels after prolonged fasting, these data suggest enhanced gluconeogenesis in PTGLKO compared with WT mice. Glucose levels in refed PTGLKO mice were higher than those in WT mice, as PTG deletion impaired glucose incorporation into glycogen and thus led to higher glucose in circulation (Figure 3B). Serum glucagon and insulin levels were comparable in WT and PTGLKO mice (Supplemental Figure 2, H and I), suggesting that the differences in glucose metabolism observed in PTGLKO mice were not a result of systemic changes in hormone levels.

To determine directly whether glycogen levels also regulate gluconeogenesis in vivo, we performed a pyruvate tolerance test in WT and PTGLKO mice. PTGLKO mice showed increased glucose production after pyruvate injection (Figure 3C). We compared the gluconeogenic gene expression levels in fasted and fed WT and PTGLKO mice and found that PTGLKO mice indeed showed higher expression of these genes when fasted (Figure 3D). Moreover, glucagon injection into mice fasted for 1 hour induced higher gluconeogenic gene expression in PTGLKO compared with WT mice (Figure 3E). Collectively, these data indicate that depletion of glycogen in PTGLKO livers upregulates gluconeogenic gene expression in vivo in response to nutritional or hormonal cues.

We also tested whether glycogen accumulation suppressed gluconeogenesis in vivo by overexpressing PTG in the liver using AAV8-PTG with AAV8-GFP as control. As previously shown (14), overexpression of PTG increased hepatic glycogen storage without affecting glucagon levels (Supplemental Figure 2, J and K). In agreement with what we observed in sgPYGL hepatocytes, gluconeogenic gene expression was also suppressed in PTG-overexpressing hepatocytes when challenged with glucagon (Supplemental Figure 2L). In direct contrast to what was observed in PTGLKO mice, overaccumulation of hepatic glycogen by PTG overexpression significantly suppressed gluconeogenesis (Figure 3F). Similarly, sgPYGL mice also showed blunted gluconeogenesis in the pyruvate tolerance test assay compared with sgNT control mice (Figure 3G). Together, these data suggest that glycogen levels per se determine the amplitude of gluconeogenesis to guarantee that glucose synthesis is suppressed during feeding and increased when glycogen levels are low during fasting.

### AMPK senses hepatic glycogen levels and positively regulates gluconeogenic gene expression.

**Figure 4:**
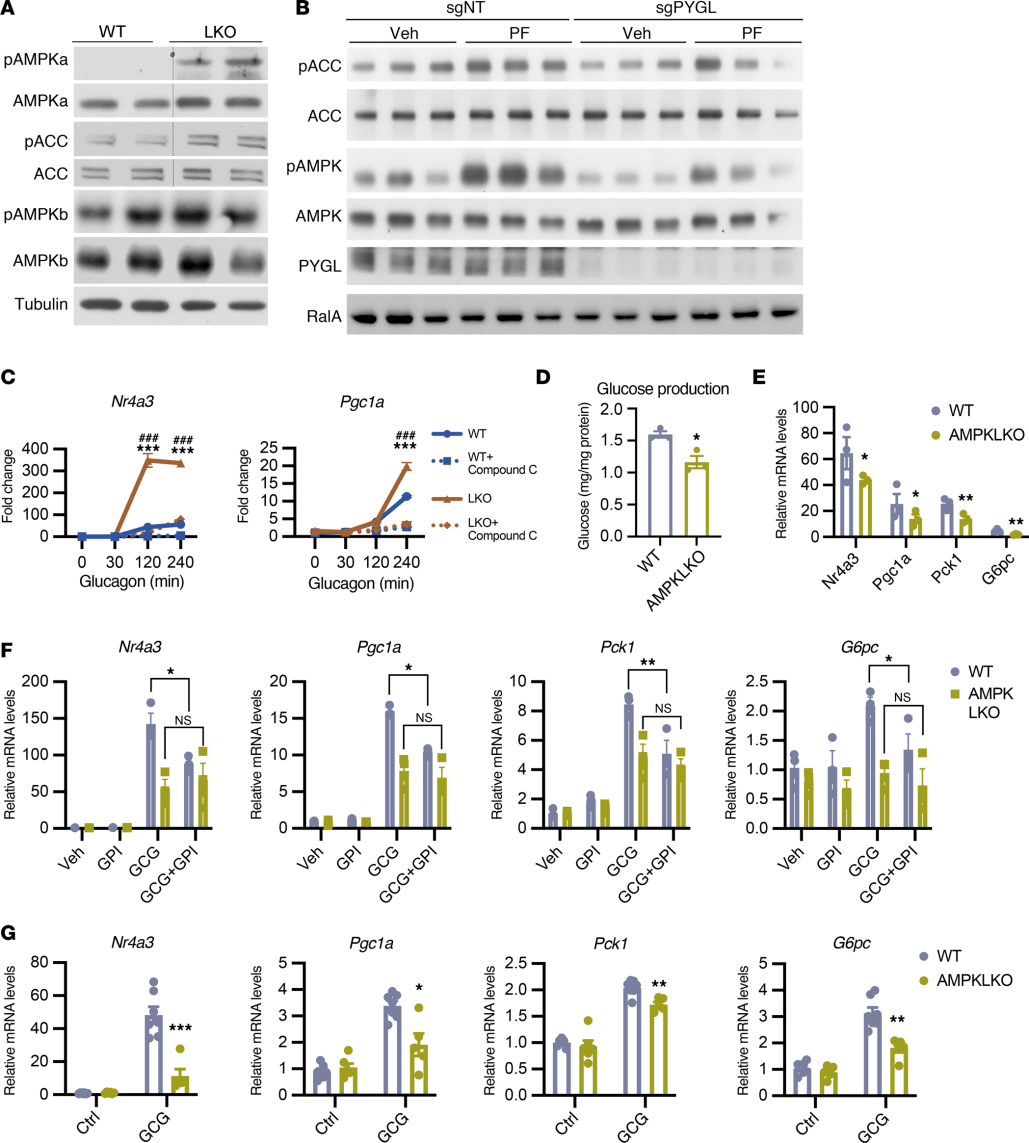
AMPK activation promotes gluconeogenic gene expression when glycogen levels are low. (**A**) Western blots of WT and PTGLKO hepatocytes. (**B**) Western blots of sgNT and sgPYGL hepatocytes. (**C**) Gene expression in WT and PTGLKO hepatocytes pretreated with vehicle or compound C followed by glucagon treatment for indicated times. (**D**) Glucagon-stimulated glucose production in WT and liver-specific AMPKα1/α2 knockout (AMPKLKO) hepatocytes. (**E**) Gene expression in WT and AMPKLKO hepatocytes treated with glucagon for 4 hours. Data were normalized to vehicle-treated group shown in Supplemental Figure 3G. (**F**) Gene expression in WT and PTGLKO hepatocytes pretreated with vehicle or GPI followed by vehicle or glucagon treatment. (**G**) Gene expression in WT and AMPKLKO liver lysates. The mice were injected with saline or 0.5 mg/kg glucagon. *n* = 3–7 per group. Experiments were performed at least 3 times. *P < 0.05; **P < 0.01; ***P < 0.001 by unpaired Student’s *t* test. ^###^*P* < 0.001 by unpaired Student’s *t* test for LKO and LKO + compound C. *** is for WT and LKO comparison.

Our data suggest that rather than acting in a parallel fashion, glycogenolysis precedes gluconeogenesis such that glycogen levels closely tune gluconeogenic activity to ensure appropriate hepatic glucose output. We next sought to identify the molecular sensor for hepatocellular glycogen levels. It is well established that glycogen directly interacts with a carbohydrate-binding domain in the β subunit of the major cellular energy sensor AMPK to reduce the activity of this enzyme (23–28). Consistent with this notion, PTGLKO hepatocytes that had reduced glycogen levels exhibited higher phosphorylation of AMPKα and its substrate acetyl-CoA carboxylase (ACC), suggesting higher AMPK activity in glycogen-depleted cells (Figure 4A and Supplemental Figure 3A). Total and phosphorylated AMPKβ (Ser^108^) levels were not altered (Figure 4A). In contrast, primary hepatocytes from sgPYGL mice showed blunted AMPK phosphorylation in comparison with sgNT cells with or without treatment with the AMPK activator PF-739 (Figure 4B and Supplemental Figure 3B).

AMPK activity is subjected to multiple regulatory mechanisms in the liver (29). To exclude other possible modes of regulation, we examined the expression of LKB1 and CaMKII, which are well-known upstream regulators of AMPK activity (30). We also assessed the binding of endogenous AMPK to PP2A, its main regulatory phosphatase (31), as well as LKB1 and CaMKII. No obvious changes were observed in total LKB1, total and phosphorylated CaMKII, and the association between AMPK and these proteins in WT and PTGLKO liver and hepatocyte lysates (Supplemental Figure 3, C and D). Thus, while we cannot completely rule out other potential mechanisms of AMPK regulation in hepatocytes, these data indicate that the altered AMPK activity in these cells was modulated primarily by glycogen levels.

To explore further the role of AMPK in mediating the impact of glycogen levels on gluconeogenic gene expression, we pretreated WT and PTGLKO hepatocytes with the AMPK inhibitor compound C, followed by treatment with glucagon. Compound C blocked over 90% of the induction of gluconeogenic genes in PTGLKO hepatocytes (Figure 4C). Because Ulk1 is a bona fide downstream substrate of AMPK, we also tested the effect of an Ulk1 inhibitor in primary hepatocytes (32). Unlike what was observed with compound C, the Ulk1 inhibitor did not suppress the expression of gluconeogenic genes in PTGLKO hepatocytes (Supplemental Figure 3E). To confirm the role of AMPK in mediating glycogen-dependent changes in gluconeogenic gene expression and gluconeogenesis, we examined primary hepatocytes isolated from WT mice and mice with liver-specific knockout of AMPKα1/α2 as an orthogonal approach to the use of compound C (AMPKLKO) (33). Glucagon-stimulated hepatocellular glucose production was blunted in AMPKLKO hepatocytes, while no change in basal activity was observed (Figure 4D and Supplemental Figure 3F). The expression of gluconeogenic genes was also decreased in AMPKLKO hepatocytes in response to glucagon treatment (Figure 4E and Supplemental Figure 3G). Moreover, treatment with GPI to increase glycogen levels decreased gluconeogenic gene expression only in WT but not AMPKLKO hepatocytes, providing further evidence to demonstrate the requirement for AMPK in mediating the control of gluconeogenic gene expression by glycogen levels (Figure 4F).

To confirm these findings in vivo, we injected WT and AMPKLKO mice with glucagon and analyzed gluconeogenic gene expression in the liver lysates. Similar to what we observed in hepatocytes, the induction by glucagon of hepatic *Nr4a3*, *Pgc1a*, *Pck1*, and *G6pc* was repressed in AMPKLKO compared with WT mice (Figure 4G). Together, these results suggest that AMPK senses hepatic glycogen levels, and mediates the increased expression of gluconeogenic genes when glycogen levels are low.

The role of AMPK in the regulation of hepatic glucose metabolism in vivo is controversial (34–37). Pyruvate tolerance tests in WT and AMPKLKO mice revealed no differences between these mice, consistent with previous findings (Supplemental Figure 3H) (37). Glucose levels were comparable between WT and AMPKLKO mice at 0, 6, 24, and 48 hours of fasting, while AMPKLKO mice exhibited lower glucose levels after 72 hours of fasting (Supplemental Figure 3I). Ketone bodies and glycerol levels were also lower in AMPKLKO mice during prolonged fasting compared with WT mice, confirming the role of AMPK in control of fatty acid oxidation (Supplemental Figure 3, J and K). We note that hepatic glucose production is under the complex regulation of gene transcription, substrate availability, and redox state (38), and our data indicate a role for AMPK only in glucagon-stimulated gluconeogenic gene expression. Reduced fatty acid oxidation may compensate for the repression of gluconeogenic gene expression in AMPKLKO mice, thus producing no net changes in hepatic glucose production in vivo, confounding the interpretations of these data. However, the reduction in serum glucose after prolonged fasting in AMPKLKO mice no doubt reflects an important role for the kinase in control of gluconeogenic gene expression.

### AMPK phosphorylates and stabilizes the transcriptional coactivator CRTC2 to promote gluconeogenesis.

**Figure 5:**
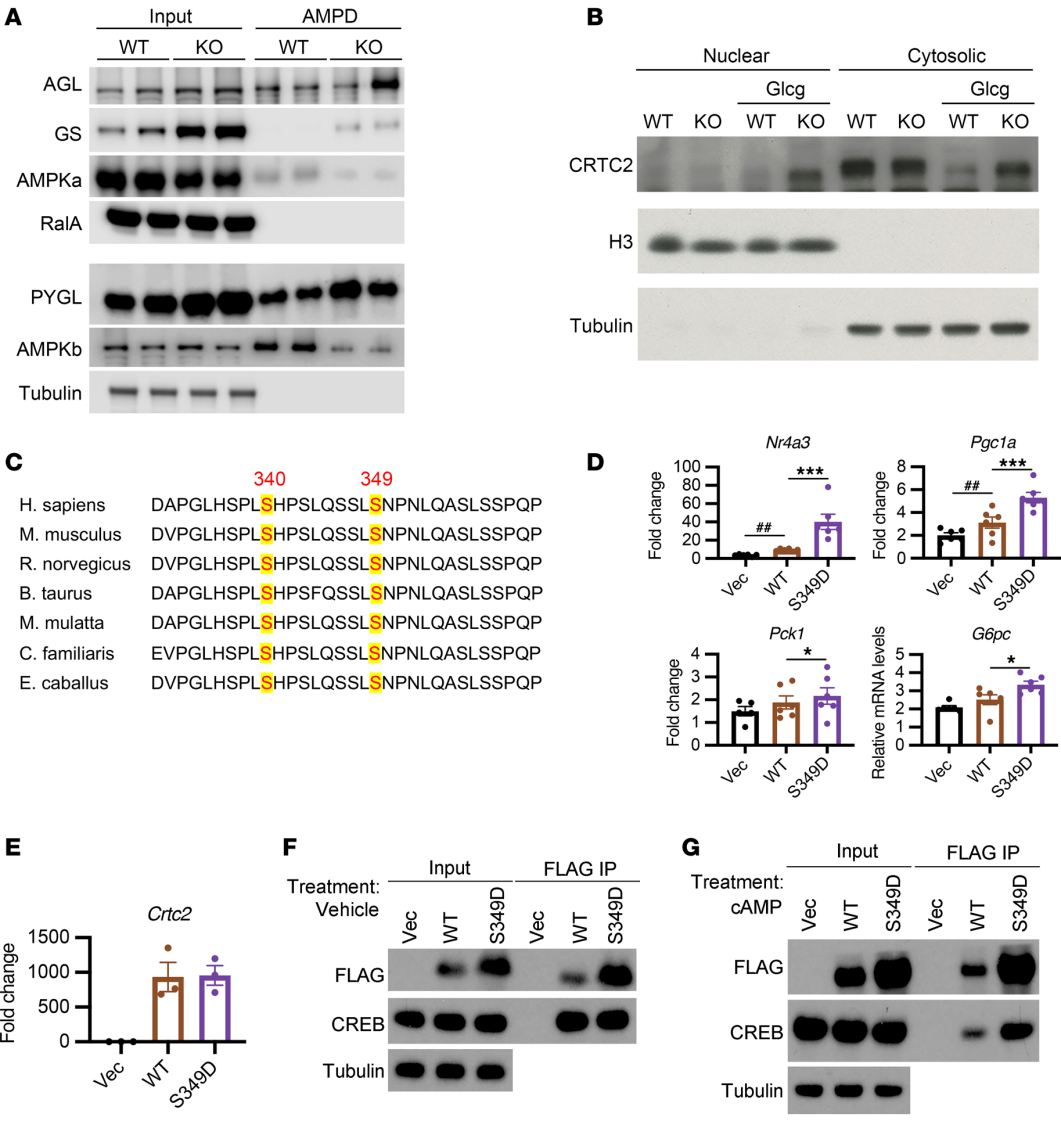
Phosphorylation of CRTC2 by AMPK promotes gluconeogenic gene expression. (**A**) Total and glycogen-bound (AMPD fraction) proteins in WT and PTGLKO primary hepatocytes. Blot quantifications are shown in Supplemental Figure 4A. (**B**) Nuclear and cytosolic proteins from WT and PTGLKO hepatocytes treated with or without glucagon. Histone H3 and tubulin were used as markers for nuclear and cytosolic fractions. (**C**) Conserved sequence of CRTC2 across species. Serine 340 and 349 are highlighted. (**D**) Gluconeogenic gene expression in AML12 cells treated with cAMP. AML12 cells were transfected with vector control, WT, or S^349^D CRTC2. Data were normalized to vehicle-treated vector group shown in Supplemental Figure 4G. (**E**) *Crtc2* gene expression in transfected AML12 cells. (**F** and **G**) Western blots of immunoprecipitated (IP) FLAG-CRTC2 and inputs. AML12 cells were transfected with FLAG-tagged indicated plasmids and treated with vehicle (**F**) or cAMP (**G**) for 1 hour. Cells were lysed to harvest input and IP proteins. *n* = 3–6 per group. Experiments were performed at least 3 times. ^#^*P < 0.05; ^##^**P < 0.01; ^###^***P < 0.001 by 1-way ANOVA. Hatch marks indicate comparison with vector group; asterisks indicate comparison with WT CRTC2.

Studies have suggested that in addition to direct inhibition, glycogen binding creates a reservoir of AMPK that might limit its accessibility to substrate, but also stabilize the protein (39–41). As we observed a higher overall AMPK activity in PTGLKO hepatocytes with lower activity in sgPYGL or GPI-treated hepatocytes, we evaluated whether the physical interaction between AMPK and liver glycogen was altered under these conditions. We performed a glycogen amylose pull-down assay (AMPD) in primary hepatocytes isolated from WT and PTGLKO mice. PYGL and the glycogen debranching enzyme AGL were pulled down equally in WT and PTGLKO hepatocytes, serving as the positive control for this assay (Figure 5A and Supplemental Figure 4A). Interestingly, we found less AMPKα and AMPKβ bound to the glycogen particle in PTGLKO hepatocytes, whereas the total levels of these proteins remained unchanged (Figure 5A and Supplemental Figure 4A). In agreement with our previous observation (14), total GS and glycogen-bound GS were increased in PTGLKO hepatocytes, indicating a possible compensatory mechanism in these mice (Figure 5A and Supplemental Figure 4A). These results indicate that abundant glycogen may sequester AMPK through a physical interaction, and the depletion of glycogen releases this inhibition to fully activate AMPK for energy production.

To determine how AMPK regulates gluconeogenic gene expression in the liver, we first examined the components of the PKA/CREB pathway by examining the phosphorylation of CREB on Ser^113^ in response to glucagon in hepatocytes derived from PTGLKO, AMPKLKO, and their control littermates. There were no differences observed in PKA signaling or CREB phosphorylation between the genotypes (Supplemental Figure 4, B and C), suggesting that CREB itself was not a target of AMPK or glycogen, and further that AMPK must be working downstream of or in parallel to CREB to control gluconeogenic gene expression. CREB target gene activation requires its binding to CRTC2, and the localization and activity of this coactivator are controlled by phosphorylation (9). We isolated the cytosolic and nuclear fractions of WT and PTGLKO hepatocytes and found that CRTC2 protein expression was increased in both fractions of PTGLKO hepatocytes without significant change in translocation (Figure 5B and Supplemental Figure 4D). CRTC2 expression correlates with energy status and determines the amplitude of gluconeogenesis (11, 12). No changes in *Crtc2* mRNA expression were observed in WT and PTGLKO hepatocytes treated with or without glucagon (Supplemental Figure 4E). We thus focused on posttranslational modifications of CRTC2. CRTC2 contains conserved canonical AMPK phosphorylation sites, including Ser^340^ and Ser^349^ (Figure 5C). These sites are highly likely to be phosphorylated by AMPK according to peptide sequence analysis (Scansite; https://scansite4.mit.edu/#home). Additionally, Ser^349^ was suggested as an AMPK substrate (42). To confirm these phosphorylation sites on CRTC2, we immunoprecipitated overexpressed CRTC2 proteins from AML12 and HEK293T cells followed by mass spectrometry analysis. Phosphorylation of Ser^349^ of CRTC2 was significantly enriched by the treatment of both cell lines with a specific AMPK activator, PF-739 (PF), while Ser^340^ phosphorylation was moderately enhanced in HEK293T cells (Supplemental Figure 4F). We therefore focused on the Ser^349^ site of CRTC2. To determine the function of Ser^349^ phosphorylation, we introduced a phospho-mimetic mutation to CRTC2 at Ser^349^. We transfected AML12 cells with WT and mutant CRTC2 before treatment with 8-Br-cAMP or vehicle. Expression of S^349^D CRTC2 increased the expression of gluconeogenic genes compared with expression of WT CRTC2 only in cells treated with 8-Br-cAMP (Figure 5D and Supplemental Figure 4G). RNA levels of WT and mutated *Crtc2* were comparable (Figure 5E). However, we observed a dramatic increase in protein levels of the S^349^D mutant (Figure 5F). Coimmunoprecipitation of CRTC2 showed similar CREB binding with the WT and mutant under basal conditions (Figure 5F). After treatment with 8-Br-cAMP, expression of the S^349^D mutant led to more CREB binding, which likely explains the increase in gluconeogenic gene expression (Figure 5G).

**Figure 6:**
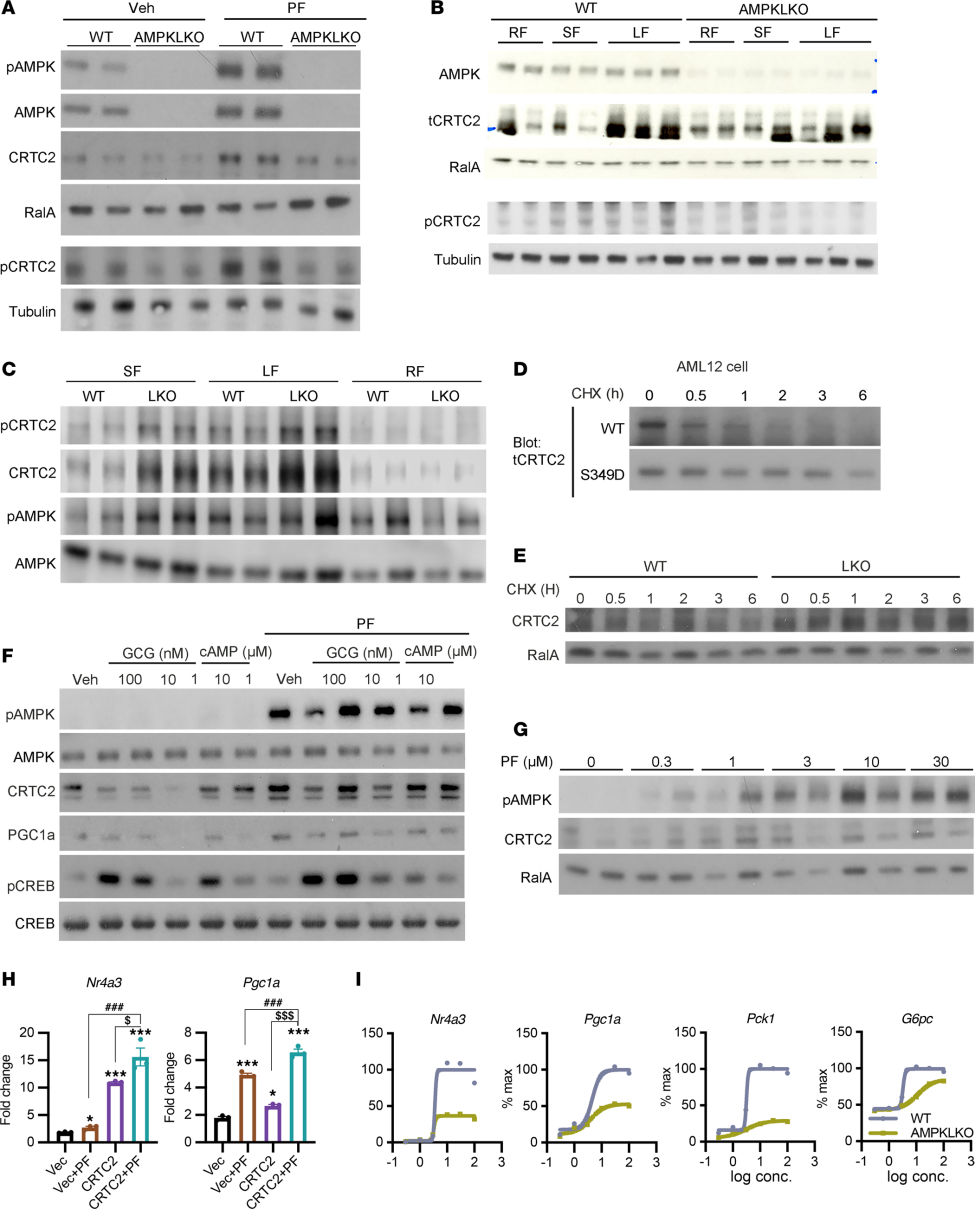
AMPK stabilizes CRTC2 and increases CRTC2 protein abundance. (**A**) Western blots of WT and AMPKLKO hepatocytes treated with vehicle or the AMPK activator PF-739 (PF). (**B**) Western blots of WT and AMPKLKO liver lysates under refeeding (RF), short-fasting (SF), and long-fasting (LF) conditions. (**C**) Western blots of WT and PTGLKO liver lysates under SF, LF, and RF conditions. (**D**) Western blots of AML12 cell lysates. Cells were transfected with WT or S^349^D CRTC2 and treated with cycloheximide (CHX) for indicated times. (**E**) Western blots and quantification of proteins from WT and PTGLKO primary hepatocytes. Cells were treated with CHX for indicated times. RalA was used as the control. (**F**) Western blots of WT primary hepatocytes treated with different doses of glucagon or cAMP. Cells were isolated from C57BL/6J mice and pretreated with vehicle or PF for 2 hours followed by glucagon or cAMP treatment for 15 minutes. (**G**) Western blots of WT hepatocytes treated with different doses of PF. (**H**) Gene expression of *Nr4a3* and *Pgc1a* in AML12 cells treated with cAMP. AML12 cells were transfected with vector or CRTC2 and pretreated with vehicle or PF. Data were normalized to vehicle-treated vector group shown in Supplemental Figure 5C. (**I**) Dose-response curve for the effect of cAMP on gluconeogenic gene expression in WT and AMPKLKO primary hepatocytes. *n* = 3 per group. Experiments were performed at least 3 times. ^#^*^$^*P* < 0.05; ^##^**^$$^*P* < 0.01; ^###^***^$$$^*P* < 0.001 by 1-way ANOVA and unpaired Student’s *t* test. * indicates comparison with vector; # indicates comparison with vector + PF; $ indicates comparison with CRTC2.

We generated an antibody that specifically recognizes the phosphorylation of CRTC2 at Ser^349^. Treatment of WT but not AMPKLKO hepatocytes with PF increased the phosphorylation of AMPK Thr^172^ and CRTC2 Ser^349^, thus confirming the necessity of AMPK for the phosphorylation of this site (Figure 6A and Supplemental Figure 5A). PF also increased the levels of CRTC2 total protein only in WT hepatocytes (Figure 6A and Supplemental Figure 5A). To understand the physiological relevance of the Ser^349^ site on CRTC2, we analyzed WT and AMPKLKO mice under short-fasting (SF; 4 hours), long-fasting (LF; 18 hours), and refeeding (RF; 3 hours of refeeding after LF) conditions. Both the phosphorylation of CRTC2 on Ser^349^ and total protein levels were increased by fasting in WT but not AMPKLKO livers (Figure 6B). In contrast, PTGLKO livers showed higher phosphorylated and total CRTC2 levels during fasting (Figure 6C). We hypothesized that phosphorylation of CRTC2 at Ser^349^ might affect protein stability to promote CREB activity and thus gluconeogenic gene expression. We thus treated WT and S^349^D CRTC2–transfected cells with cycloheximide to determine the half-life of CRTC2. The protein stability of the mutant form was dramatically increased compared with WT (WT, τ_1/2_ = 1.3 hours; S^349^D, τ_1/2_ = 5.3 hours) (Figure 6D). Similarly, endogenous CRTC2 also showed increased stability in PTGLKO compared with WT primary hepatocytes (Figure 6E and Supplemental Figure 5B). To confirm the effect of AMPK activation on CRTC2 protein levels, we pretreated WT primary hepatocytes with PF followed by glucagon or 8-Br-cAMP. PF treatment increased AMPK phosphorylation and resulted in an overall increase in CRTC2 protein levels and subsequent PGC1a protein expression without affecting phospho-CREB (Figure 6F). The effect of PF on CRTC2 protein levels was dose dependent (Figure 6G and Supplemental Figure 5C).

To examine the functional impact of the AMPK/CRTC2 signaling axis, we tested how overexpression of CRTC2 and AMPK activation affected the expression of gluconeogenic genes. While PF treatment and CRTC2 overexpression alone both increased *Nr4a3* and *Pgc1a* expression, the combination of the two showed an additive effect in inducing gluconeogenic gene expression (Figure 6H and Supplemental Figure 5D). Together, these data suggested that AMPK activation increases CRTC2 protein abundance, which in turn amplifies gluconeogenic gene transcription. To confirm this, we examined whether AMPK deletion shifted the dose response to cAMP in primary hepatocytes. Without affecting the EC_50_, AMPK deletion in hepatocytes led to dramatically decreased maximal gene induction by cAMP stimulation (Figure 6I and Supplemental Figure 5E). Collectively, these data show that once activated by low glycogen levels, AMPK phosphorylates CRTC2 at Ser^349^ to stabilize the protein, thus increasing the amplitude of hepatocellular gluconeogenic gene expression in response to glucagon.

## Discussion

**Figure 7:**
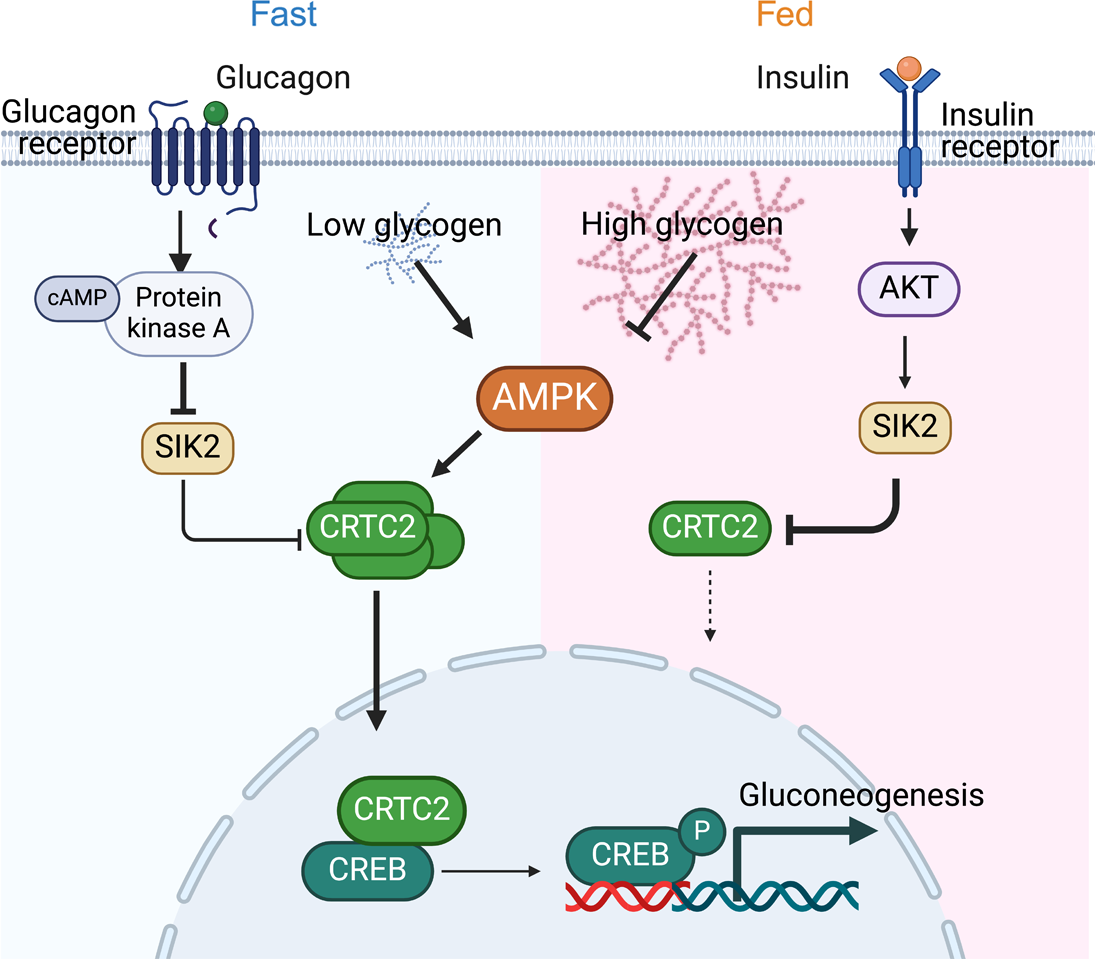
Working model. Glycogen levels tune gluconeogenesis in response to nutritional and hormonal cues. Under fasting or glucagon stimulation, decrease in glycogen levels activates AMPK, which phosphorylates and stabilizes CRTC2, increasing its abundance to prime hepatocytes for gluconeogenesis. cAMP inhibits SIK2 activity, permitting the translocation of CRTC2 into the nucleus. The binding of CRTC2 to CREB induces gluconeogenic gene expression. Under fed conditions or insulin stimulation, glycogen accumulation suppresses AMPK activity. Meanwhile, the activation of AKT increases SIK2 activity to sequester CRTC2 in the cytosol. Thus, glycogen levels ensure efficient glucose output during energy shortage and suppress glucose production during energy surplus. This figure was created in BioRender (https://BioRender.com/q31x887).

Hepatic glycogen is the first choice for glucose storage and mobilization in fed and fasted states. While glycogenolysis fuels hepatic glucose output during the initial stages of fasting, gluconeogenesis reaches its peak after glycogen is depleted (43, 44). Indeed, there have been hints of molecular links between glycogenolysis and gluconeogenesis. Liver glycogen levels and gluconeogenesis are coordinately regulated in insulin-resistant people with obesity (45). Gluconeogenesis is increased in livers of mice lacking liver glycogen synthase (46), and glycogen phosphorylase inhibition is associated with reduced gluconeogenesis (19). To our knowledge, our findings provide the first mechanistic link for these two processes: we show here that hepatic glycogen levels regulate gluconeogenic gene expression through a newly defined glycogen/AMPK/CRTC2 axis (Figure 7). This mechanism provides an additional layer of regulation to ensure appropriate glucose production during periods of either energy shortage or surplus in an efficient and economical manner in which hepatic glucose metabolism is sequentially ordered by hormonal and nutritional status.

While glycogen has long been viewed as a mere reservoir for energy, our findings that glycogen levels closely guide liver gluconeogenesis in a cell-autonomous fashion indicate a function beyond energy storage. Glycogen depletion via PTG knockout increased expression of gluconeogenic genes in response to glucagon, while glycogen accumulation by PTG overexpression, glycogen phosphorylase knockdown, or pharmacological inhibition decreased expression of these genes. These are not the first data to suggest a signaling role for glycogen. We recently showed that glycogen accumulation and turnover is crucial for the beiging process in white adipose tissue (47). Brain glycogen contributes to aging, locomotion, and memory formation (48–50). Liver glycogen accumulation is reported to be a key step in the oncogenic initiation of hepatocellular carcinoma (51). Moreover, glycogen accumulation is associated with the development of fatty liver disease (52), and PTG deletion can reverse this state (14). Collectively, these studies showcase multiple mechanisms by which glycogen acts as a metabolic messenger to actively regulate cellular processes beyond energy storage.

While it is clear that glycogen levels are sensed differently in varied cell types as an indication of overall energy status, we sought to understand how hepatocytes sense glycogen to control expression of gluconeogenic genes. Numerous studies have shown that AMPK serves as a vital cellular stress and energy shortage sensor (23, 30, 53–55). In addition to responding to the canonical pathway through changes in AMP/ATP ratio, AMPK also senses the availability of other nutrients, including glycogen (40). AMPK contains a carbohydrate-binding module on its regulatory β subunit that interacts with glycogen when it is appropriately branched, resulting in inhibition of the enzyme both in vivo and in cell-free assays (23–25, 56, 57). Moreover, it is well known that AMPK activation upregulates *Pgc1a*, a transcriptional cofactor for HNF4 and FOXO1 that serves as a master regulator of both mitochondrial biogenesis and gluconeogenesis (17, 58, 59). Indeed, we found that AMPK activation promotes gluconeogenic gene expression during fasting, while targeted AMPK deletion or inhibition by a small molecule reduced gluconeogenic gene expression and glucose production in primary hepatocytes and shifted the dose-response curve to cAMP treatment. These data strongly suggest that AMPK senses glycogen levels and translates these to regulation of gluconeogenic gene expression. We also note that glucagon may indirectly drive AMPK activity over the longer term as a result of its stimulation of glycogenolysis. Additionally, AMPK directly phosphorylates and inhibits glycogen synthase (60), suggesting a positive-feedback loop for glycogen’s regulation of AMPK.

Interestingly, a previous report on liver-specific glycogen synthase knockout (LGSKO) mice found no major difference in AMPK expression and activity in the knockout livers except after prolonged fasting, although gluconeogenic gene expression was elevated, as seen in PTGLKO mice (46). Although we cannot precisely account for these differences, we note that LGSKO mice were completely devoid of liver glycogen, whereas PTGLKO mice retain 50% liver glycogen under ad libitum feeding conditions. These differences are reflected in markedly distinct parameters of systemic glucose metabolism, as LGSKO mice show glucose intolerance and elevated ketone bodies and triglyceride levels, traits not seen in PTGLKO mice. It is also not known whether glucose 6-phosphate levels, ADP/ATP ratio, and other metabolites are different between LGSKO and PTGLKO mice. We speculate that the total lack of glycogen in LGSKO mice might lead to compensatory changes in metabolism and cellular stress response to maintain ATP levels and thus keep AMPK activity low. Another possibility is that the suppression of AMPK activity by glycogen may also require the scaffolding function of PTG. Indeed, previous studies have shown a direct interaction between AMPK and PTG (61, 62).

How do glycogen levels influence AMPK activity? Numerous studies have demonstrated that glycogen directly binds to AMPK through its β subunit, resulting in reduced catalytic activity (23–28). However, a recent study using a whole-body knockin mutation of AMPKβ subunits that cannot bind to glycogen reported that AMPK-glycogen binding stabilized AMPK (26, 41). We did observe that fewer AMPKα and AMPKβ subunits were bound to glycogen in PTGLKO hepatocytes. However, this reduction in glycogen-bound AMPK was not accompanied by a change in total AMPK protein levels in whole-cell lysates, indicating that glycogen does not play a major role in net AMPK protein stabilization during energy shortage. Additionally, mice with liver-specific deletion of glycogen synthase, which completely lack hepatic glycogen, had no reduction in AMPK protein levels (46). It is possible that the mutations of AMPKβ render the enzyme less stable independently of its glycogen binding activities. However, in agreement with the previously hypothesized reciprocal regulation between AMPK and glycogen (40), we speculate that glycogen binding may provide a reservoir of AMPK protein, sequestering its availability and activity in the glycogen compartment, which is then released for activation upon glycogenolysis. Whether other signals or changes in nutritional state are required for this activation is unknown.

Interestingly, AMPK is generally thought to inhibit gluconeogenesis, a contention largely based on the beneficial glucoregulatory effects of the AMPK activator metformin in diabetic mice (34, 63–65). However, this conclusion has been challenged by several findings, including the discovery of other metformin targets (36, 66, 67), the AMPK-independent effect of metformin on hepatic gluconeogenesis (37, 68), and our finding that treatment with an AMPK activator stabilizes CRTC2 and induces hepatic *Pgc1a* and *Nr4a3* expression, which contributes to enhanced gluconeogenesis in the presence of glucagon receptor activation. While the transcriptional impact of AMPK is clearly pro-gluconeogenic, it is also known that AMPK modulates other aspects of hepatic glucose metabolism, including metabolic flux and reprogramming, hepatokine production, and inflammation, to improve overall glucose homeostasis, especially in the short term (33, 69). Indeed, AMPKLKO mice showed no difference in pyruvate tolerance and fasting glucose levels in comparison with WT littermates, confirming previous findings that AMPK deletion did not significantly change glucose levels in mice that had not been subject to fasting in vivo (35–37). However, 72 hours of fasting revealed lower blood glucose levels in knockout mice, confirming the role of AMPK in preserving gluconeogenic gene expression under these conditions. Taken together, these data paint a complex picture of AMPK’s role in hepatic metabolism in which the enzyme responds to low energy states by increasing energy utilization, while providing glucose for other tissues to utilize during prolonged fasting through increased gluconeogenic gene expression.

In our search for an AMPK substrate that might influence gluconeogenic gene expression, we first considered the master early gene regulator CREB. However, neither manipulation of glycogen levels nor AMPK activity had any effect on glucagon- or cAMP-stimulated CREB phosphorylation. We then turned our attention to the CREB coactivator CRTC2 and identified two novel AMPK consensus phosphorylation sites that affect protein stability and thus levels. While CREB is a direct target of PKA, CRTC2 is a critical coactivator that separately relays cytosolic energy status to initiate appropriate glucose production. Deletion of CRTC2 reduced the transcriptional response to fasting in hepatocytes without affecting short-term glucose homeostasis in vivo (70). This phenomenon is similar to what we and others observed in AMPKLKO mice, where cellular glucose production is reduced and whole-body glucose metabolism is unaffected, which is largely due to compensatory pathways in both the liver and other tissues. Phosphorylation of CRTC2 by SIK2 in the fed state initiates binding to 14-3-3, retention of the protein in the cytoplasm, and ultimately degradation (10). In the fasted state, SIK2 is inhibited, and whereas activated AMPK has no effect on the SIK2 sites within CRTC2, it phosphorylates Ser^349^, stabilizing the protein and permitting nuclear localization for coactivation with CREB, consistent with the phenotypes observed (71). Indeed, the phosphorylation of CRTC2 by both AMPK and SIK2 kinases subjects the protein to multiple additional posttranslational modifications, including acetylation, ubiquitination, and *O*-GlcNAcylation (9). The targets of the CREB:CRTC2 complex are also of great interest in understanding the control of energy metabolism by the glycogen/AMPK axis. Our data suggest that *Nr4a3* and *Pgc1a* are key targets that ultimately regulate the expression of genes encoding gluconeogenic enzymes. We previously reported that global knockout of PTG produced dramatic upregulation of PGC1α/PPARα target genes that may be beneficial in obesity (14). Further structure/activity analyses will be required to understand how these posttranslational modifications control CRTC2 stability, localization, and activity to modulate energy metabolism.

The elucidation of the glycogen/AMPK/CRTC2 signaling pathway may potentially provide new therapeutic approaches for metabolic diseases. GPI was previously developed as a candidate to lower blood glucose levels by blocking glycogen breakdown (19). However, a major side effect of these compounds was excessive lipid accumulation in the liver. It is possible that GPI-induced glycogen accumulation suppresses AMPK activity, which simultaneously dampens gluconeogenesis and lipid catabolism, while increasing lipogenesis. Our finding may also provide an explanation for the therapeutic benefits of glucagon receptor agonists for individuals with obesity and type 2 diabetes reported recently in clinical trials (72, 73). A low-glycogen, high-AMPK profile in hepatocytes promotes gluconeogenic gene expression and fatty acid oxidation, while inhibiting lipogenesis. Considering that the downstream signaling pathways of glucagon predominantly enhance catabolic metabolism, the activation of AMPK by lowering of glycogen levels is likely to further sensitize cells to glucagon receptor agonists, potentiating their beneficial metabolic effect. These possibilities will require further investigation.

## Methods

### Sex as a biological variable.

Our study examined male and female animals, and similar findings are reported for both sexes.

### Animal studies.

PTG-floxed mice and AMPKα1/α2-floxed mice were generated in-house and crossed with *Alb-Cre* mice (26, 33). C57BL/6J (catalog 000664) and spCas9 knockin mice (catalog 026175) were acquired from The Jackson Laboratory. AAV8-Ppp1r3c virus (VectorBuilder) and AAV8-EGFP virus (VectorBuilder) were administered at 2 × 10^11^ genome copies per mouse through tail vein injection. AAVs expressing single guide RNA (sgRNA) targeting PYGL were generated in-house. AAVs expressing non-targeting sgRNA were purchased from Vector Laboratories. Injected mice were allowed to recover for a week before any procedures. For glucagon injections, mice were fasted for 1 hour before the injection of 0.5 mg/kg glucagon intraperitoneally. Mice were sacrificed 30 minutes after the injection to harvest liver tissues. All experiments were done with wild type and their respective knockout littermates. All mice were kept on a 12-hour light, 12-hour dark cycle. All mice had free access to food and water. Both sexes were included in the experiments, and the age of the mice used is specified in the text. Mice were fasted for 6 hours before sacrifice, and tissues were collected for protein, RNA, and glycogen extraction and histology analysis.

### Metabolic cage studies.

Metabolic cage study was carried out with the Promethion multiplexed metabolic measurement system (Sable Systems International) in a temperature-controlled cabinet. Mice were single-housed for 5 days and acclimated in metabolic chambers for 3 days before the measurement of gas exchange, food intake, and ambulatory activity.

### Metabolic assays.

Body composition was measured with the EchoMRI system. For pyruvate tolerance tests, mice fasted for 6 hours were injected intraperitoneally with 1.5 g of sodium pyruvate (Sigma-Aldrich) per kilogram of body weight. Blood glucose level was measured by tail vein bleeding at 0, 15, 30, 45, 60, 90, and 120 minutes after the injection using a Nova Max glucometer (Nova Biomedical).

### Histology.

Mouse livers were dissected and fixed in 4% paraformaldehyde for 48 hours and then washed and stored in 70% ethanol until processing. Periodic acid–Schiff stains were performed by the Tissue Technology core facility at UCSD Moores Cancer Center.

### Glycogen extraction.

Glycogen extraction was done as previously described (14, 47). Samples were boiled for 30 minutes in 500 μL of 30% KOH solution with constant shaking. One hundred microliters of 1 M NaSO_4_ was added, followed by the addition of 1.2 mL pure ethanol to all samples. Samples were then boiled for 5 minutes and centrifuged for 5 minutes at 13,000*g*. Pellets were washed 3 times by resuspension of the pellet in 500 μL of double-distilled water and then 1 mL of pure ethanol. After the last wash, pellets were allowed to completely air-dry and then resuspended in 50 mM sodium acetate (pH 4.8) containing 0.3 mg/mL amyloglucosidase and incubated at 37°C overnight. Autokit glucose assay (Fuji) was used to determine the amount of glycogen by comparing to a glycogen standard curve.

### ELISA and metabolite measurements.

Plasma samples were collected with EDTA-coated capillaries, centrifuged at 2,000*g* for 20 minutes, and stored at –80°C until measurement. Assays were performed according to the manufacturers’ protocol for glucagon ELISA (Mercodia), insulin ELISA (Crystal Chem), amino acid assays (Cayman Chemical), non-esterified free fatty acid (Thermo Fisher Scientific), glycerol (Thermo Fisher Scientific), and β-hydroxybutyrate (Cayman Chemical).

### Primary hepatocyte isolation and cell culture.

Mice were anesthetized and perfused with 15 mL calcium-free HEPES–phosphate buffer (pH 7.4) followed by 25 mL HEPES–phosphate buffer (pH 7.4) containing 40 μg/mL Liberase TM (Roche). The last perfusion was done with 25 mL calcium-free HEPES–phosphate buffer (pH 7.4). Primary hepatocytes were mechanically dissociated from the perfused liver in 30 mL cold HEPES–phosphate buffer (pH 7.4) and passed through a 70 μm mesh nylon filter (Corning). Hepatocytes were spun down at 50*g* for 5 minutes. The supernatant was discarded, and the pellet was resuspended in 50 mL HEPES–phosphate buffer (pH 7.4) containing 36% Percoll and spun at 100*g* for 10 minutes. The pellet was resuspended in 10 mL pre-warmed William’s E medium (Life Technologies) supplemented with 10% FBS, 10 mM HEPES buffer, 2 mM l-glutamine, 8 mg/L gentamicin, antibiotic antimycotic solution, 1 μM dexamethasone, 4 μg/mL insulin, and 1 mM glucose. All hepatocytes were plated in collagen-coated plates at 4 × 10^5^ per milliliter. Four hours after plating, fresh William’s E medium was replaced to remove unattached and dead cells. For all treatment with glucagon and cAMP, hepatocytes were fasted for 1 hour in William’s E medium supplemented with 10 mM HEPES buffer, 2 mM l-glutamine, 8 mg/L gentamicin, SPA, 1 μM dexamethasone, and 1 mM glucose (FBS-free medium). Treatments were also performed in this FBS-free medium. AML12 and HEK293T cell lines were purchased from ATCC (stock CRL-2254 and CRT-3216, respectively) and cultured in DMEM-F12 or DMEM medium supplemented with 10% FBS and 1% penicillin/streptomycin. Transfection was performed with Lipofectamine 3000 (Thermo Fisher Scientific). DMEM-F12 medium, FBS, William’s E medium, HEPES buffer, glutamine stock solution, SPA, penicillin/streptomycin, and gentamicin were from Gibco. Dexamethasone, insulin, and glucose were purchased from Sigma-Aldrich.

### Glucose production assay.

Primary hepatocytes were fasted in FBS-free medium for 1 hour and then switched to no-glucose medium supplemented with 20 mM sodium lactate, 2 mM sodium pyruvate, and 2 mM glutamine and treated with glucagon for 4 hours. Culture medium and cell lysates were collected, and glucose production was determined with Autokit glucose assay (Fuji) with data normalized to protein concentration.

### cAMP measurement.

Primary hepatocytes were stimulated with glucagon in FBS-free medium for 5 or 15 minutes. The reaction was ended by removal of the culture medium and addition of 0.1 M HCl to lyse the cells. Cellular cAMP levels were determined with the cAMP enzyme immunoassay kit (Sigma-Aldrich), and data were normalized to total protein concentration.

### Real-time PCR.

Total RNA was extracted from cells or tissues with TRIzol reagent and PureLink RNA purification kit (Invitrogen). Complementary DNA was synthesized from total RNA with HiScript III supermix (Vazyme) and amplified with SYBR Green Supermix (Vazyme) using an Applied Biosystems QS5 real-time PCR System. All data were normalized to the expression of 36b4. Sequences of primers used are listed in Supplemental Table 1.

### Western blotting and immunoprecipitation.

Liver tissues or cells were lysed in buffer containing 1% Nonidet P-40, 150 mM NaCl, 0.1 mM EDTA, 50 mM Tris-HCl, and Halt proteinase and phosphatase inhibitor cocktail (Thermo Fisher Scientific). Protein quantification was done using the DC protein assay (Bio-Rad). Nuclear and cytosolic cell fractions were isolated as previously described (3). FLAG immunoprecipitation (IP) assays were carried out by incubation of cell lysates with magnetic FLAG beads (Sigma-Aldrich) in the PP1 homogenization buffer containing 50 mM HEPES (pH 7.2), 2 mM EDTA, 0.2% β-mercaptoethanol, and 2 mg/mL glycogen (2). IP products were washed 3 times and boiled in 2× SDS buffer. Endogenous IP assays were carried out by incubation of cell lysates with AMPKα primary antibody overnight at 4°C and then with magnetic protein A/G beads (Thermo Fisher Scientific) at room temperature for another 2 hours. IP products were washed 3 times and prepared in 2× SDS buffer. AMPD assays were carried out as described earlier (14). Equal amounts of protein lysates were resolved on SDS-PAGE gels and transferred to the nitrocellulose membrane. The membranes were blocked in 5% BSA and incubated with various primary antibodies overnight at 4°C. After 3 washes, the membranes were incubated with peroxidase-conjugated secondary antibodies for 1 hour and visualized with ECL chemiluminescent substrate (Thermo Fisher Scientific).

### Mass spectrometry.

FLAG-tagged WT and S^349^D CRTC2 was transfected to AML12 or HEK293T cells and immunoprecipitated with magnetic FLAG beads (Sigma-Aldrich). Lysates were resolved on SDS-PAGE gels, and corresponding bands were excised after silver staining of the gel (Thermo Fisher Scientific). Bands were digested with chymotrypsin, and liquid chromatography–mass spectrometry was performed by the UCSD Biomolecular and Proteomics Mass Spectrometry core. Data analysis was performed with PEAKS Studio (Bioinformatics Solutions).

### Plasmids.

pcDNA-FLAG-CRTC2 plasmid was purchased from Addgene (22975) and cloned into the pLVX-CMV-3XFLAG vector. Site-directed mutagenesis was performed with the In-Fusion snap assembly master mix (Takara). The following primers were used for site-directed mutagenesis: S^349^D 5′-CTCCCTAGACAATCCCAACCTCCAGGCTTCC-3′, and 5′-GGATTGTCTAGGGAGGACTGCAGGGATG-3′. The sequence of all mutated plasmids was verified.

### Antibodies and reagents.

Phospho-AMPKα (Thr^172^; 2531), AMPKα (5831), phospho-AMPKβ (Ser^108^; 4181), AMPKβ (4150), phospho-ACC (Ser^79^; 3661), ACC (3676), phospho-CREB (Ser^133^; 9198), CREB (9197), PKA substrate (9621), GS (3886), LKB1 (3050), phospho-CaMKII (Thr^286^; 12716), CaMKII (3362), β-tubulin (2128), and histone H3 (9715) antibodies were purchased from Cell Signaling. Phospho-CRTC2 (Ser^349^) antibody was customized by AB Clonal against synthetic peptide QSSL(S-p)NPNL. FLAG antibody was purchased from Sigma-Aldrich (F3165). PGC1a antibody was from EMD Millipore (AB3242). RalA antibody was purchased from BD Biosciences (610221). Glucagon receptor antibody was purchased from Thermo Fisher Scientific (702602). β-Actin antibody was from Santa Cruz Biotechnology (SC-47778). PYGL (15851-1-AP) and AGL (16582-1-AP) antibodies were from Proteintech. CRTC2 antibody was provided by Marc Montminy’s laboratory (Salk Institute, San Diego, California, USA). Glucagon, 8-Br-cAMP, glycogen, cycloheximide, sodium pyruvate, sodium lactate, and glutamine were purchased from Sigma-Aldrich. Glycogen phosphorylase inhibitor (CP-91149) and compound C were purchased from Cayman Chemical. PF-739 and Ulk1 inhibitor (SBI-0206965) were purchased from MedChem Express.

### Statistics.

Results are presented as mean ± SEM. The comparisons were carried out using 2-tailed unpaired Student’s *t* test or 1-way ANOVA followed by Tukey-adjusted multiple comparisons. Data were plotted with GraphPad Prism. *P* values less than 0.05 were considered significant. Statistical tests used are stated in the figure legends.

### Study approval.

All animal use was approved by the Institutional Animal Care and Use Committee at the University of California, San Diego.

### Data availability.

All data are available in the main text or in the Supporting Data Values file.

## Author contributions

ARS supervised the project. BZ and ARS conceptualized the project, acquired funding, and wrote the manuscript. BZ, MMJ, TY, TNN, LHM, SX, and BD performed the experiments. JO, FY, AMX, and JEP provided the AAV for sgPYGL.

## Supplementary Material

Supplemental data

Unedited blot and gel images

Supporting data values

## Version 1 06/02/2025

Electronic publication

## References

[1] Ekberg K (1999). Contributions by kidney and liver to glucose production in the postabsorptive state and after 60 hours of fasting. Diabetes.

[2] Brady MJ (1997). Role of protein targeting to glycogen (PTG) in the regulation of protein phosphatase-1 activity. J Biol Chem.

[3] Printen JA (1997). PTG, a protein phosphatase 1-binding protein with a role in glycogen metabolism. Science.

[4] Berman HK (1998). Overexpression of protein targeting to glycogen (PTG) in rat hepatocytes causes profound activation of glycogen synthesis independent of normal hormone- and substrate-mediated regulatory mechanisms. J Biol Chem.

[5] Dornbos P (2022). Evaluating human genetic support for hypothesized metabolic disease genes. Cell Metab.

[6] Unni U (2023). 1580-P: role of hepatic glycogen on nocturnal gluconeogenesis in type 2 diabetes. Diabetes.

[7] Montminy MR, Bilezikjian LM (1987). Binding of a nuclear protein to the cyclic-AMP response element of the somatostatin gene. Nature.

[8] Conkright MD (2003). TORCs: transducers of regulated CREB activity. Mol Cell.

[9] Altarejos JY, Montminy M (2011). CREB and the CRTC co-activators: sensors for hormonal and metabolic signals. Nat Rev Mol Cell Biol.

[10] Screaton RA (2004). The CREB coactivator TORC2 functions as a calcium- and cAMP-sensitive coincidence detector. Cell.

[11] Koo SH (2005). The CREB coactivator TORC2 is a key regulator of fasting glucose metabolism. Nature.

[12] Liu Y (2008). A fasting inducible switch modulates gluconeogenesis via activator/coactivator exchange. Nature.

[13] Brady MJ (1997). The regulation of glycogen synthase by protein phosphatase 1 in 3T3-L1 adipocytes. Evidence for a potential role for DARPP-32 in insulin action. J Biol Chem.

[14] Lu B (2014). Metabolic crosstalk: molecular links between glycogen and lipid metabolism in obesity. Diabetes.

[15] Armstrong CG (1998). Identification of the separate domains in the hepatic glycogen-targeting subunit of protein phosphatase 1 that interact with phosphorylase a, glycogen and protein phosphatase 1. Biochem J.

[16] Pei L (2006). NR4A orphan nuclear receptors are transcriptional regulators of hepatic glucose metabolism. Nat Med.

[17] Yoon JC (2001). Control of hepatic gluconeogenesis through the transcriptional coactivator PGC-1. Nature.

[18] Capozzi ME (2022). The past, present, and future physiology and pharmacology of glucagon. Cell Metab.

[19] Martin WH (1998). Discovery of a human liver glycogen phosphorylase inhibitor that lowers blood glucose in vivo. Proc Natl Acad Sci U S A.

[20] Platt RJ (2014). CRISPR-Cas9 knockin mice for genome editing and cancer modeling. Cell.

[21] Jungas RL (1992). Quantitative analysis of amino acid oxidation and related gluconeogenesis in humans. Physiol Rev.

[22] Aikawa T (1973). Gluconeogenesis and amino acid metabolism. II. Inter-organal relations and roles of glutamine and alanine in the amino acid metabolism of fasted rats. J Biochem.

[23] McBride A (2009). The glycogen-binding domain on the AMPK beta subunit allows the kinase to act as a glycogen sensor. Cell Metab.

[24] Polekhina G (2003). AMPK beta subunit targets metabolic stress sensing to glycogen. Curr Biol.

[25] Hudson ER (2003). A novel domain in AMP-activated protein kinase causes glycogen storage bodies similar to those seen in hereditary cardiac arrhythmias. Curr Biol.

[26] Wojtaszewski JF (2002). Glycogen-dependent effects of 5-aminoimidazole-4-carboxamide (AICA)-riboside on AMP-activated protein kinase and glycogen synthase activities in rat skeletal muscle. Diabetes.

[27] Liu Y (2021). A Fbxo48 inhibitor prevents pAMPKα degradation and ameliorates insulin resistance. Nat Chem Biol.

[28] Watt MJ (2004). Beta-adrenergic stimulation of skeletal muscle HSL can be overridden by AMPK signaling. FASEB J.

[29] Townsend LK, Steinberg GR (2023). AMPK and the endocrine control of metabolism. Endocr Rev.

[30] Garcia D, Shaw RJ (2017). AMPK: mechanisms of cellular energy sensing and restoration of metabolic balance. Mol Cell.

[31] Jeon SM (2016). Regulation and function of AMPK in physiology and diseases. Exp Mol Med.

[32] Kim J (2011). AMPK and mTOR regulate autophagy through direct phosphorylation of Ulk1. Nat Cell Biol.

[33] Zhao P (2020). An AMPK-caspase-6 axis controls liver damage in nonalcoholic steatohepatitis. Science.

[34] Ma T (2022). Low-dose metformin targets the lysosomal AMPK pathway through PEN2. Nature.

[35] Cokorinos EC (2017). Activation of skeletal muscle AMPK promotes glucose disposal and glucose lowering in non-human primates and mice. Cell Metab.

[36] Madiraju AK (2014). Metformin suppresses gluconeogenesis by inhibiting mitochondrial glycerophosphate dehydrogenase. Nature.

[37] Foretz M (2010). Metformin inhibits hepatic gluconeogenesis in mice independently of the LKB1/AMPK pathway via a decrease in hepatic energy state. J Clin Invest.

[38] Petersen MC (2017). Regulation of hepatic glucose metabolism in health and disease. Nat Rev Endocrinol.

[39] Bendayan M (2009). Association of AMP-activated protein kinase subunits with glycogen particles as revealed in situ by immunoelectron microscopy. J Histochem Cytochem.

[40] Steinberg GR, Hardie DG (2023). New insights into activation and function of the AMPK. Nat Rev Mol Cell Biol.

[41] Hoffman NJ (2020). Genetic loss of AMPK-glycogen binding destabilises AMPK and disrupts metabolism. Mol Metab.

[42] Sonntag T (2017). Analysis of a cAMP regulated coactivator family reveals an alternative phosphorylation motif for AMPK family members. PLoS One.

[43] Cahill GF (1976). Starvation in man. Clin Endocrinol Metab.

[44] Perry RJ (2018). Leptin mediates a glucose-fatty acid cycle to maintain glucose homeostasis in starvation. Cell.

[45] Flatt JP (1996). Glycogen levels and obesity. Int J Obes Relat Metab Disord.

[46] Irimia JM (2010). Impaired glucose tolerance and predisposition to the fasted state in liver glycogen synthase knock-out mice. J Biol Chem.

[47] Keinan O (2021). Glycogen metabolism links glucose homeostasis to thermogenesis in adipocytes. Nature.

[48] Sinadinos C (2014). Neuronal glycogen synthesis contributes to physiological aging. Aging Cell.

[49] Duran J (2013). Impairment in long-term memory formation and learning-dependent synaptic plasticity in mice lacking glycogen synthase in the brain. J Cereb Blood Flow Metab.

[50] Sun RC (2021). Brain glycogen serves as a critical glucosamine cache required for protein glycosylation. Cell Metab.

[51] Liu Q (2021). Glycogen accumulation and phase separation drives liver tumor initiation. Cell.

[52] Chen J (2024). Hepatic glycogenesis antagonizes lipogenesis by blocking S1P via UDPG. Science.

[53] Pinkosky SL (2020). Long-chain fatty acyl-CoA esters regulate metabolism via allosteric control of AMPK β1 isoforms. Nat Metab.

[54] Zhang CS (2017). Fructose-1,6-bisphosphate and aldolase mediate glucose sensing by AMPK. Nature.

[55] Auciello FR (2014). Oxidative stress activates AMPK in cultured cells primarily by increasing cellular AMP and/or ADP. FEBS Lett.

[56] Koay A (2010). AMPK beta subunits display isoform specific affinities for carbohydrates. FEBS Lett.

[57] Wojtaszewski JF (2003). Regulation of 5’AMP-activated protein kinase activity and substrate utilization in exercising human skeletal muscle. Am J Physiol Endocrinol Metab.

[58] Wu Z (1999). Mechanisms controlling mitochondrial biogenesis and respiration through the thermogenic coactivator PGC-1. Cell.

[59] Puigserver P, Spiegelman BM (2003). Peroxisome proliferator-activated receptor-gamma coactivator 1 alpha (PGC-1 alpha): transcriptional coactivator and metabolic regulator. Endocr Rev.

[60] Carling D, Hardie DG (1989). The substrate and sequence specificity of the AMP-activated protein kinase. Phosphorylation of glycogen synthase and phosphorylase kinase. Biochim Biophys Acta.

[61] Vernia S (2009). AMP-activated protein kinase phosphorylates R5/PTG, the glycogen targeting subunit of the R5/PTG-protein phosphatase 1 holoenzyme, and accelerates its down-regulation by the laforin-malin complex. J Biol Chem.

[62] Solaz-Fuster MC (2008). Regulation of glycogen synthesis by the laforin-malin complex is modulated by the AMP-activated protein kinase pathway. Hum Mol Genet.

[63] Agius L (2020). The metformin mechanism on gluconeogenesis and AMPK activation: the metabolite perspective. Int J Mol Sci.

[64] Zhou G (2001). Role of AMP-activated protein kinase in mechanism of metformin action. J Clin Invest.

[65] Lochhead PA (2000). 5-Aminoimidazole-4-carboxamide riboside mimics the effects of insulin on the expression of the 2 key gluconeogenic genes PEPCK and glucose-6-phosphatase. Diabetes.

[66] Zhang SY (2023). Metformin triggers a kidney GDF15-dependent area postrema axis to regulate food intake and body weight. Cell Metab.

[67] Tobar N (2023). Metformin acts in the gut and induces gut-liver crosstalk. Proc Natl Acad Sci U S A.

[68] Foretz M (2023). Metformin: update on mechanisms of action and repurposing potential. Nat Rev Endocrinol.

[69] Kahn BB (2005). AMP-activated protein kinase: ancient energy gauge provides clues to modern understanding of metabolism. Cell Metab.

[70] Le Lay J (2009). CRTC2 (TORC2) contributes to the transcriptional response to fasting in the liver but is not required for the maintenance of glucose homeostasis. Cell Metab.

[71] Capriotti E (2005). I-Mutant2.0: predicting stability changes upon mutation from the protein sequence or structure. Nucleic Acids Res.

[72] Jastreboff AM (2023). Tirzepatide once weekly for the treatment of obesity. N Engl J Med.

[73] Rosenstock J (2023). Retatrutide, a GIP, GLP-1 and glucagon receptor agonist, for people with type 2 diabetes: a randomised, double-blind, placebo and active-controlled, parallel-group, phase 2 trial conducted in the USA. Lancet.

