# Supplementary material for: Hepatic glycogen directly regulates gluconeogenesis through an AMPK/CRTC2 axis in mice
Source: J Clin Invest. 2025 Jun 2;135(11):e188363. doi: 10.1172/JCI188363 (PMC12126231; doi:10.1172/JCI188363)
Supplement: Supplemental data [file jci-135-188363-s340.pdf]

Supplemental data for

Hepatic glycogen directly regulates gluconeogenesis through an AMPK-CRTC2 axis in mice

Authors: Bichen Zhang[1], Morgan M. Johnson[1#], Timothy Yuan[1#], Tammy-Nhu Nguyen[1#], Junichi Okada[2], Fajun Yang[2], Alus M. Xiaoli[2], Liana H. Melikian[1], Songran Xu[1], Benyamin Dadpey[1], Jeffrey Pessin[2,3], and Alan R. Saltiel[1, 4]*.

* To whom correspondence should be addressed. Email: asaltiel@ucsd.edu.

The PDF file includes:

Figures S1-S5
Table S1

Supplementary Materials

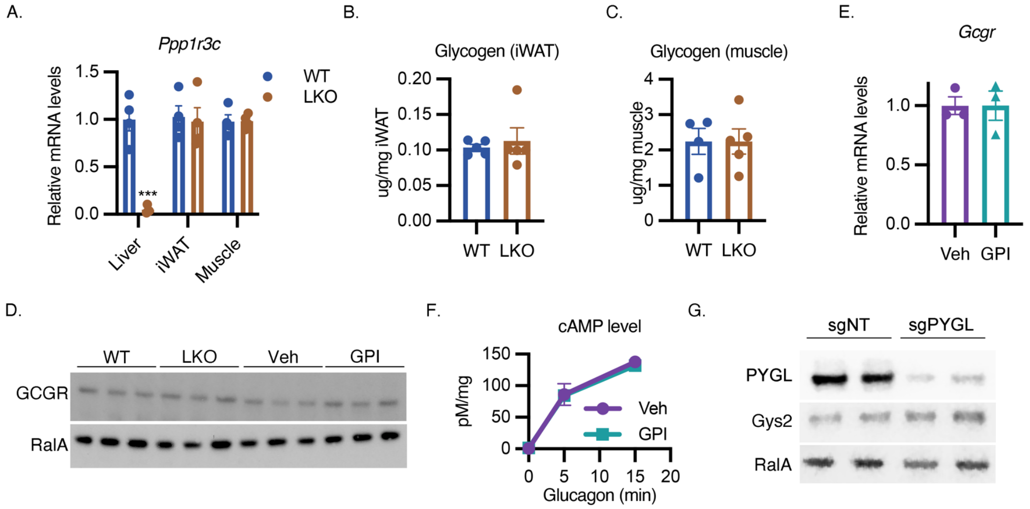

Figure S1. Specific deletion of PTG in the liver reduces hepatic glycogen levels. A, Gene expression of *Ppp1r3c* (gene encoding PTG) in the liver, inguinal white adipose tissue (iWAT), and muscle from WT and LKO mice. **B-C,** Glycogen levels in iWAT and muscle from WT and LKO mice. **D,** Western blots of hepatocytes from WT and LKO mice (left 6 lanes); and hepatocytes from C57BL/6J mice treated with vehicle or glycogen phosphorylase inhibitor (GPI) overnight. **E,** Expression of glucagon receptor (Gcgr) in vehicle (veh) and GPI-treated hepatocytes. **F,** cAMP levels in vehicle or GPI-treated hepatocytes treated with glucagon. **G,** Knockdown of Pygl in primary hepatocytes by CRISPR-mediated gene editing. n = 3-5 per group. *** $p < 0.001$ by unpaired Student's *t* test.

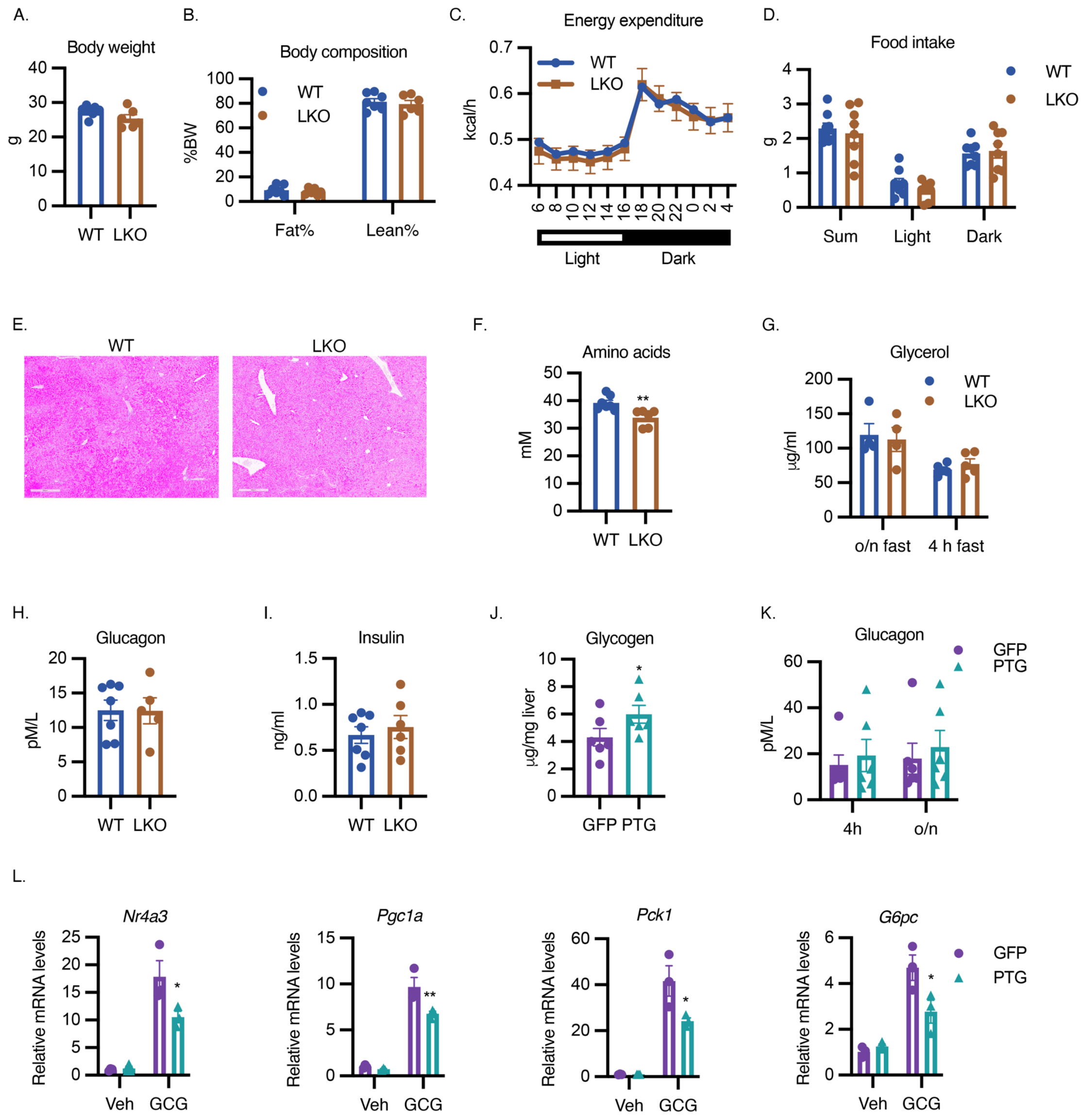

Figure S2. Physiology of WT and PTGLKO mice. All measurement from a-f were taken in 16-week-old WT and PTGLKO mice fed on chow diet. **A,** Body weight of WT and LKO mice. **B,** Body composition of WT and PTGLKO mice. **C,** Energy expenditure of WT and PTGLKO during light and dark cycles. **D**, Accumulative food intake of WT and PTGLKO during light and dark cycles. **E,** Periodic acid-Schiff (PAS) staining of WT and PTGLKO liver sections. **F,** Serum amino acid levels in WT and PTGLKO mice fasted overnight. **G,** Serum glycerol levels in WT and PTGLKO mice under fast and fed conditions. **H,** Glucagon levels in WT and PTGLKO mice fasted for 18 h. **I,** Insulin levels in refed WT and PTGLKO mice. **J,** Glycogen levels in liver lysates from mice injected with AAV8-GFP and AAV8-PTG. **K,** Glucagon levels in mice injected with AAV8-GFP and AAV8-PTG. **L,** Gluconeogenic gene expression in control and PTG overexpressed hepatocytes. n = 6-8 per group. ** $p < 0.01$ by unpaired Student's *t* test.

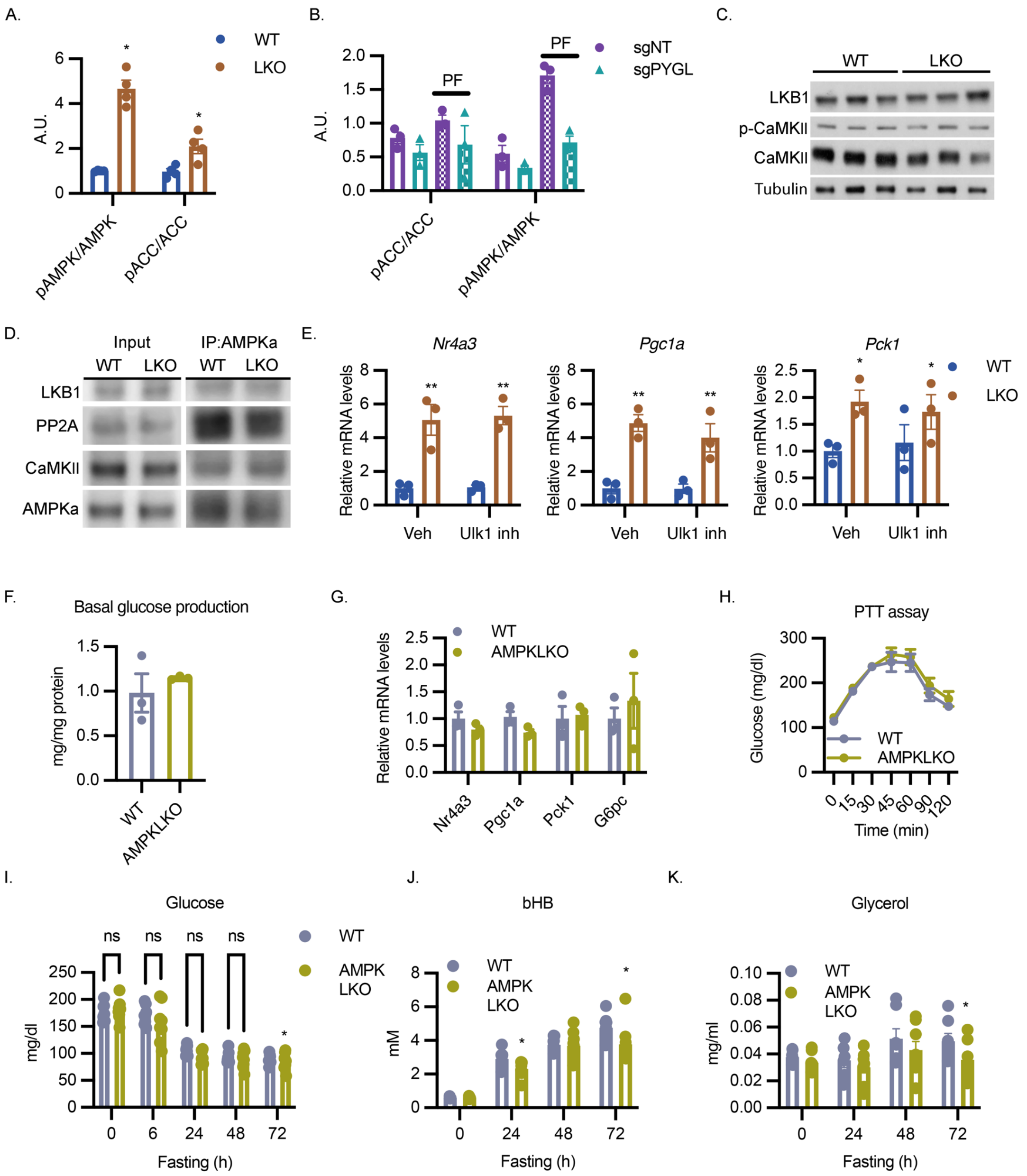

Figure S3. WT and AMPKLKO showed comparable glucose levels in vivo. A, Quantification of phospho/total AMPK and phospho/total ACC in WT and PTGLKO hepatocytes shown in Fig. 4A. **B,** Quantification of phosphor/total AMPK and ACC in sgNT and sgPYGL hepatocytes shown in Fig. 4B. **C,** Western blots of WT and PTGLKO liver lysates. **D,** Western blots of endogenous immunoprecipitation of AMPKa in WT and PTGLKO primary hepatocytes. **E,** Gluconeogenic gene expression in WT and PTGLKO cells. Primary hepatocytes were pre-treated with vehicle or the specific Ulk1 inhibitor SBI-0206965 for 1h before the treatment of glucagon for 4 h. **F,** Glucose production of WT and AMPKLKO hepatocytes under basal conditions. **G,** Gene expression in WT and AMPKLKO hepatocytes treated with vehicle for 4 h. **H,** PTT assays in WT and AMPKLKO mice. **I,** Glucose levels in WT and AMPKLKO mice fasted for 0, 6, 24, 48, and 72 h. **J,** β-hydroxybutyrate (bHB) levels in WT and AMPKLKO mice. **K,** Glycerol levels in WT and AMPKLKO mice. n = 3-8 per group. * $p < 0.05$; ** $p < 0.01$ by unpaired Student's *t* test.

A. B.

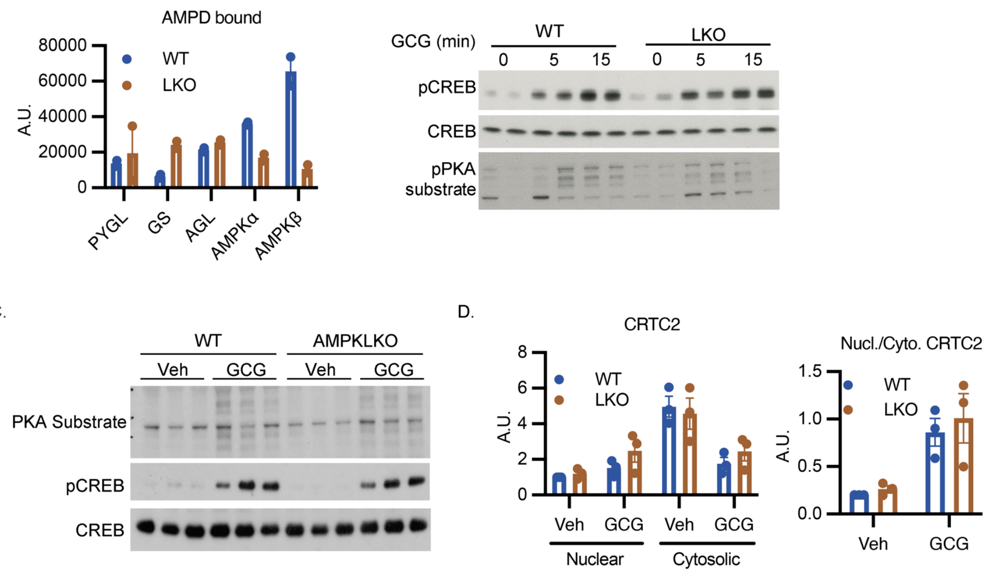

E.

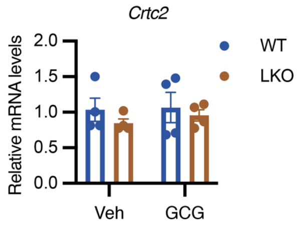

F.

Peptide sequence	AML12 cells		HEK293T cells	
	Veh	PF	Veh	PF
SHPSLQSSLS349NPNLQASL	5.23%	16.44%	0.73%	3.81%
S340HPSLQSSLSNPNLQASL	Not detected	Not detected	1.07%	2.34%

G.

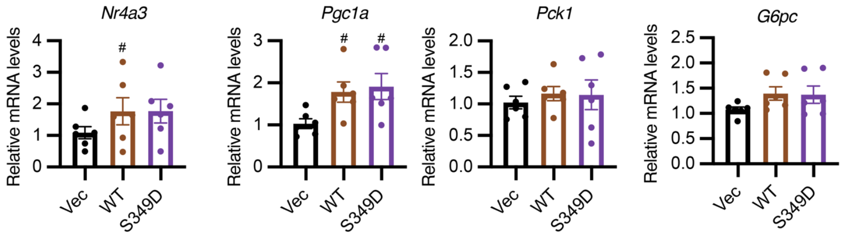

Figure S4. PKA pathway and *Crtc2* RNA expression is comparable in WT and PTGLKO hepatocytes. A, Quantification of glycogen-bound proteins (AMPD fraction) in primary hepatocytes shown in Fig. 5A. **B,** Western blots of WT and PTGLKO primary hepatocytes treated with glucagon (GCG) with indicated period. **C,** Western blots of WT and AMPKLKO hepatocytes treated with GCG for 15 min. **D,** Quantification of cytosolic and nuclear CRTC2 in WT and PTGLKO hepatocytes shown in Fig. 5B. **E,** *Crtc2* gene expression in WT and PTGLKO hepatocytes treated with or without glucagon. **F,** Percentage of modified CRTC2 in AML12 and HEK293T cells with vehicle or PF treatment. **G,** Gluconeogenic gene expression in AML12 cells treated with vehicle. AML12 cells were transfected with vector control, WT, or $S^{349}D$ CRTC2. n = 3-6. $^{\#}$ $p < 0.05$ by one-way ANOVA analysis. $^{\#}$ indicates comparison with vector group.

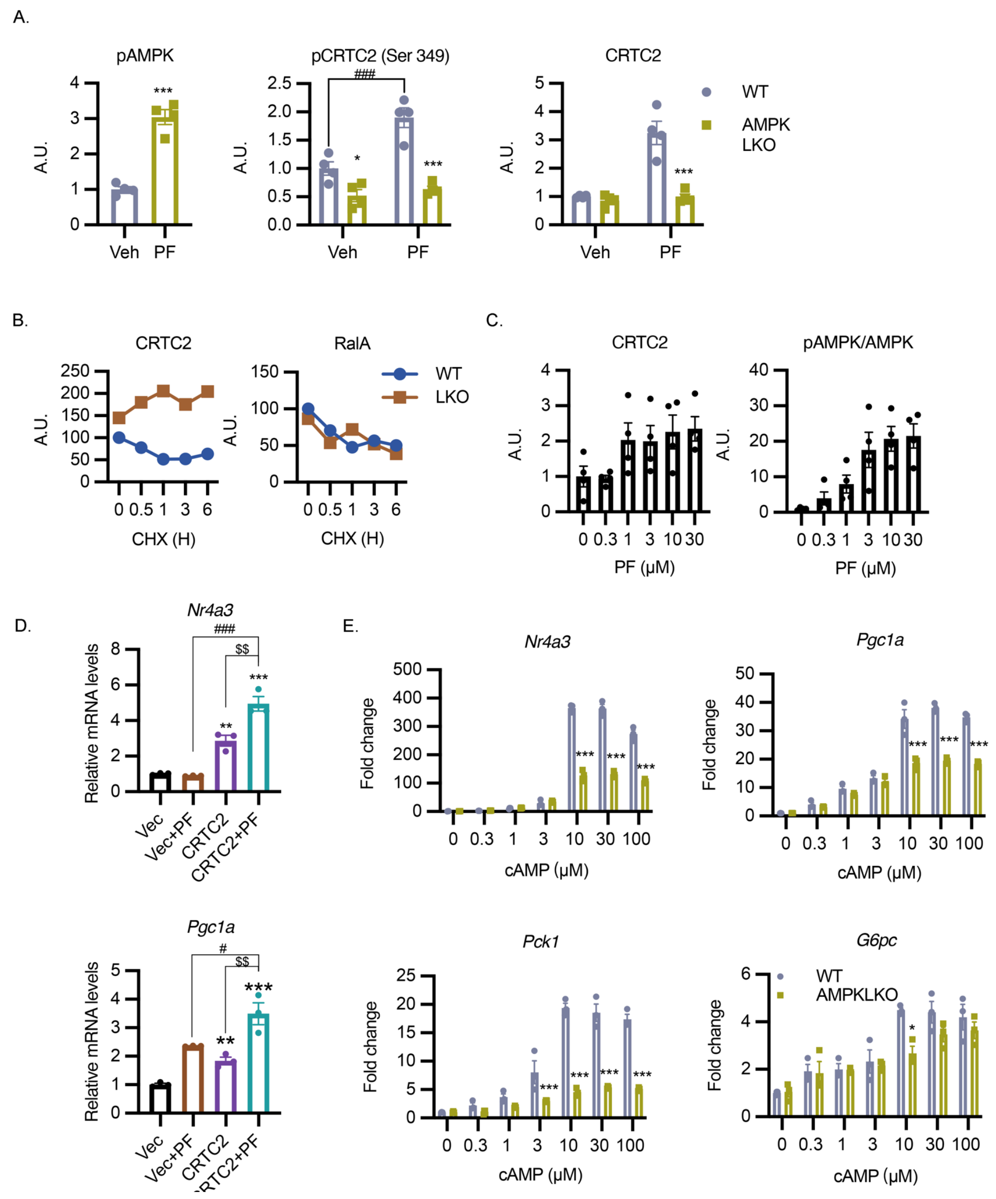

Figure S5. AMPK potentiates the maximum induction of gluconeogenic gene expression to cAMP treatment through CRTC2. A. Quantification of pCRTC2 and CRTC2 levels in WT and AMPKLKO hepatocytes, and pAMPK levels in WT hepatocytes shown in Fig. 6A. **B**. Quantification of CRTC2 protein expression levels. RalA was used as a control. **C**. Quantification of CRTC2 and pAMPK/AMPK level in WT and AMPKLKO hepatocytes shown in Fig. 6G. **D**. Gene expression of *Nr4a3* and *Pgc1a* in AML12 cells treated with vehicle. AML12 cells were transfected with vector or CRTC2 and pre-treated with vehicle. **E**. Gene expression in WT and AMPKLKO hepatocytes treated with different doses of 8-Br-cAMP. n = 3-4. $^{\#*}$ $p < 0.05$; $^{\#\#}$ $^{**\$\$}$ $p < 0.01$; $^{***\#\#\#\$\$\$}$ $p < 0.001$ by one-way ANOVA analysis and unpaired Student's *t* test.

Table S1. Sequence of qPCR primers

Name	Sequence
36B4-F	AGATGCAGCAGATCCGCAT
36B4-R	GTTCTTGCCCATCAGCACC
Ppp1r3c-F	TGATCCATGTGCTAGATCCACG
Ppp1r3c-R	ACTCTGCGATTTGGCTTCCTG
Gcgr-F	CAATGCCACCACAACCTAAGCC
Gcgr-R	GGCAGGAAATGTTGGCAGTGGT
Nr4a3-F	TGCGTGCAAGCCCAGTATAG
Nr4a3-R	ATAAGTCTGCGTGGCGTAAGT
Pgc1a-F	TATGGAGTGACATAGAGTGTGCT
Pgc1a-R	CCACTTCAATCCACCCAGAAAG
Pck1-F	ACACACACACATGCTCACAC
Pck1-R	ATCACCGCATAGTCTCTGAA
G6pc-F	CGACTCGCTATCTCGAAGTGA
G6pc-R	GTTGAACGAGTCTCCGACGA
Crtc2-F	ATGAACCCTAACCCCCAAGAC
Crtc2-R	CGTTCTCCTCAATAGCAGGGA
