# Supplementary material for: Hepatic glycogen directly regulates gluconeogenesis through an AMPK/CRTC2 axis in mice
Source: J Clin Invest. 2025 Jun 2;135(11):e188363. doi: 10.1172/JCI188363 (PMC12126231; doi:10.1172/JCI188363)

Unedited gel and blot images

Figure S1D

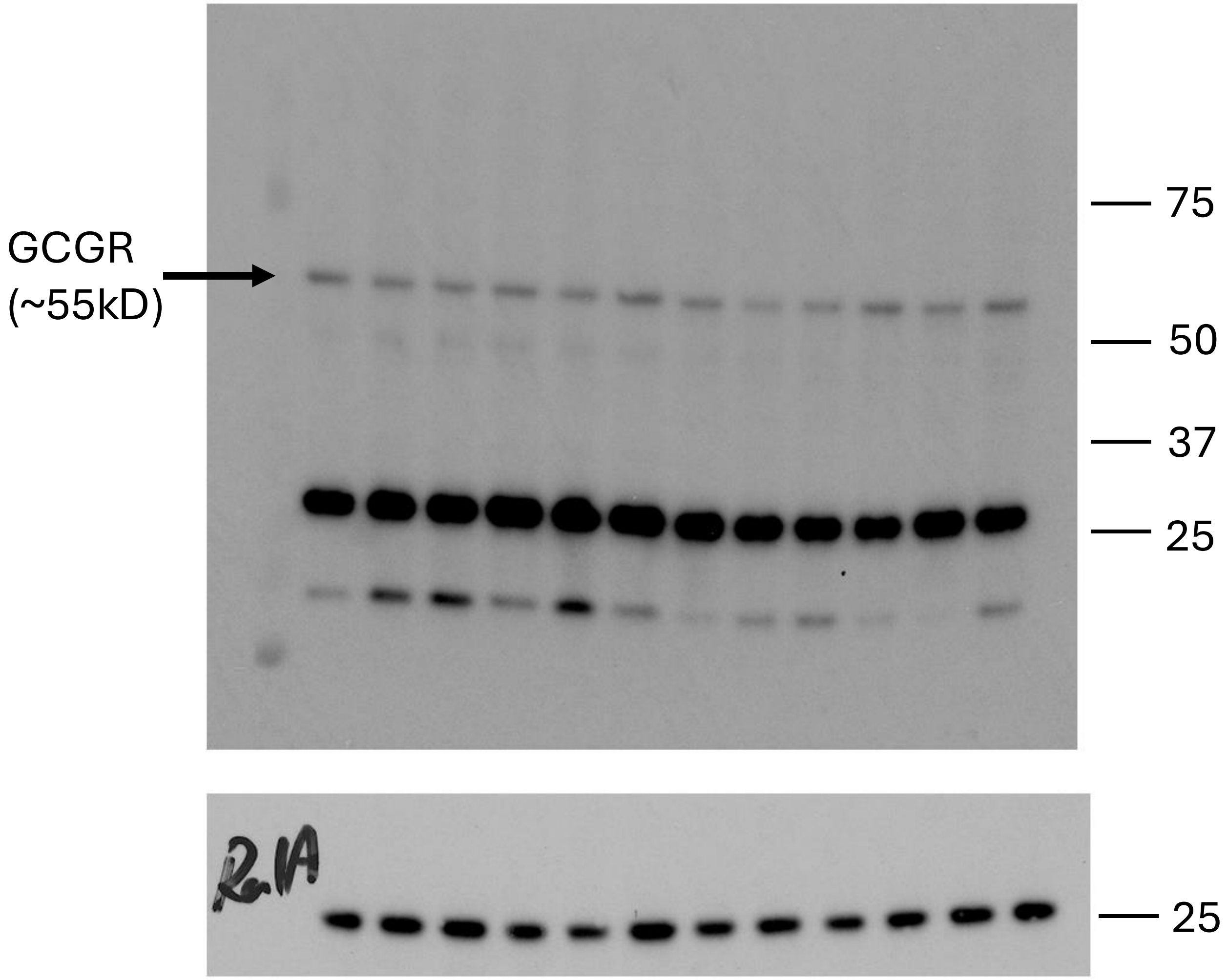

Figure S1G

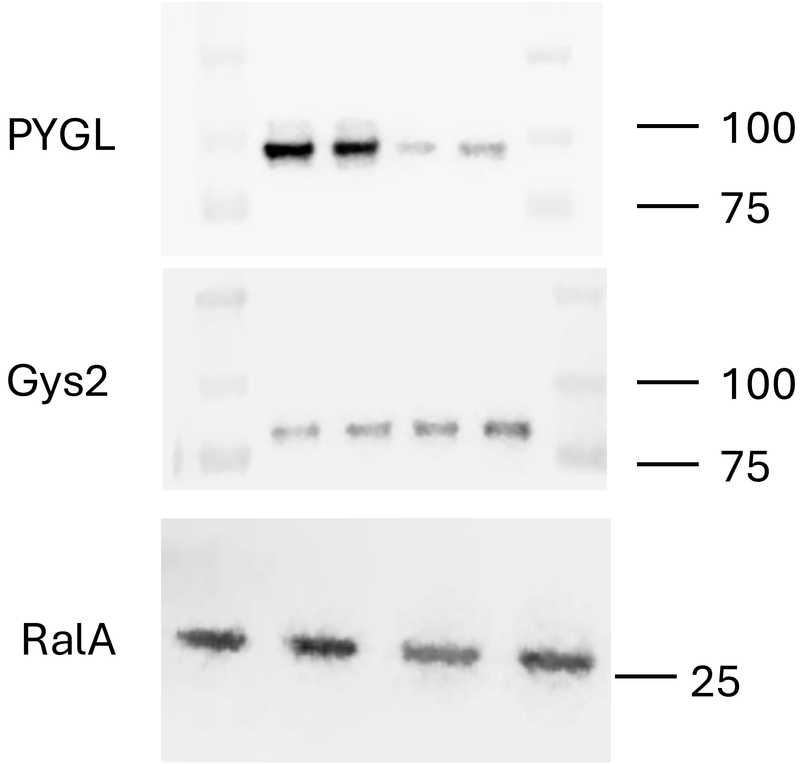

Figure S3C

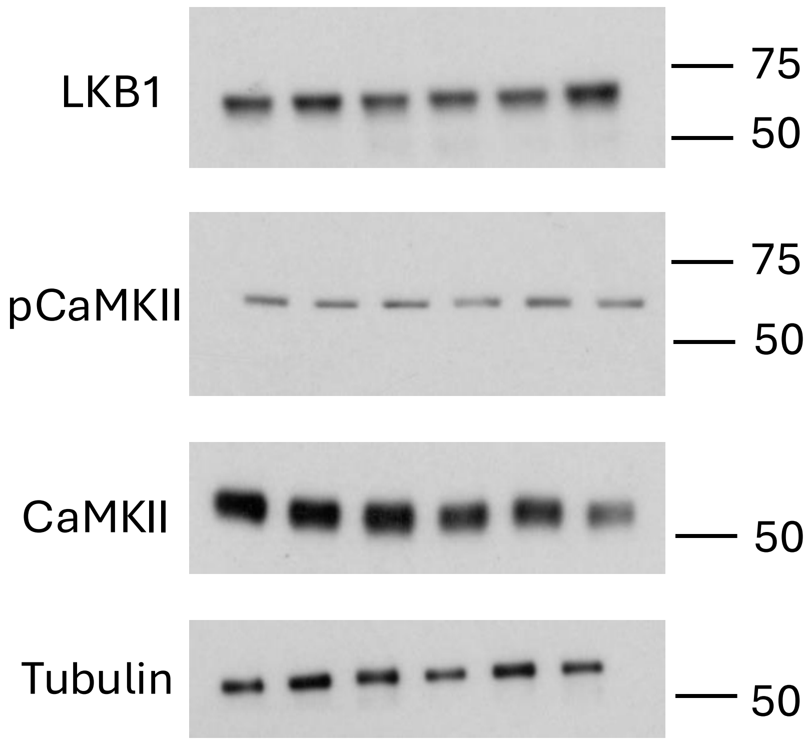

Figure S3D

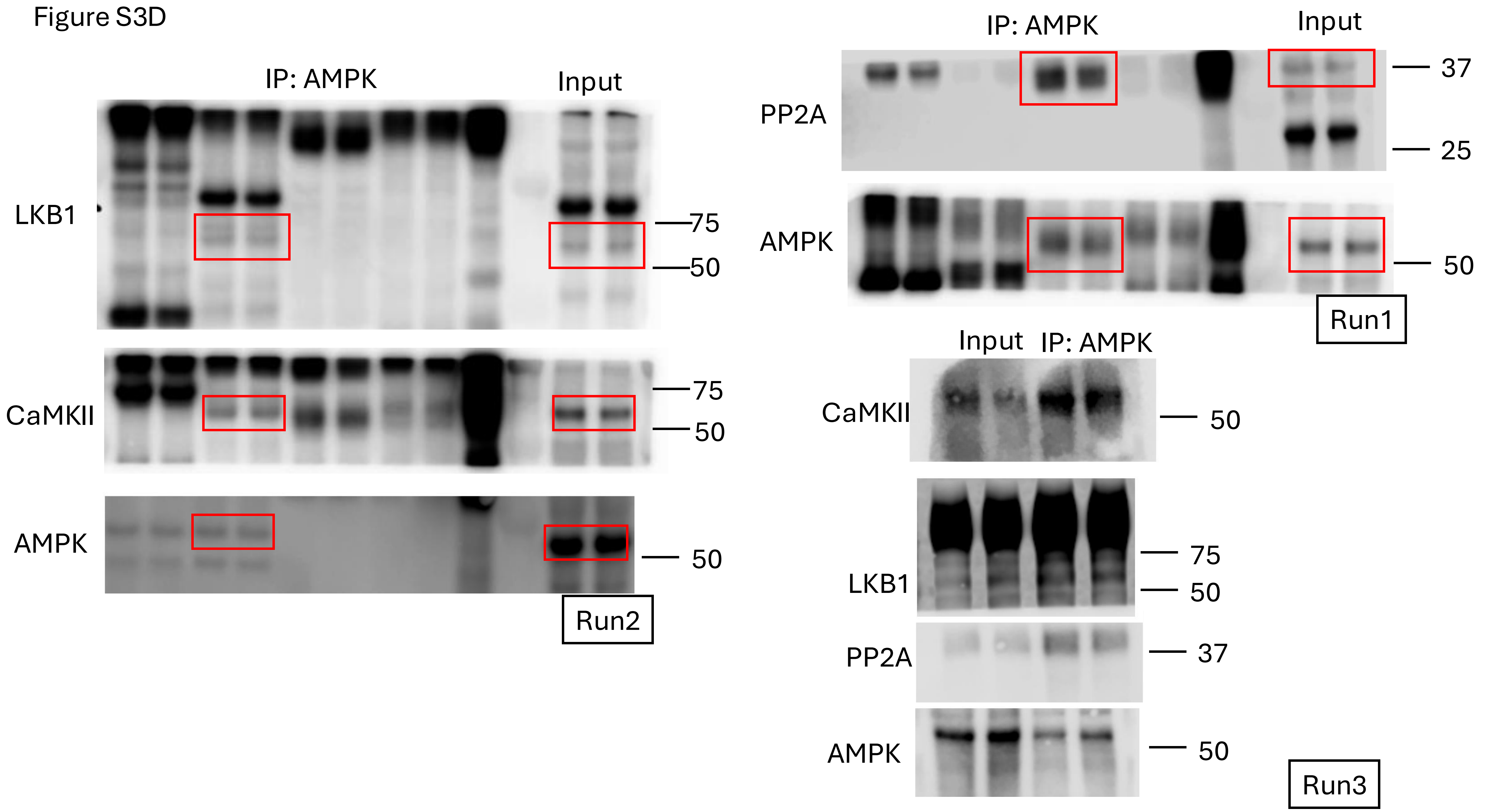

IP: AMPK
Input
LKB1
75
50
CaMKII
75
50
AMPK
50
Run2
IP: AMPK
Input
PP2A
37
25
AMPK
50
Run1
Input
IP: AMPK
CaMKII
50
LKB1
75
50
PP2A
37
AMPK
50
Run3

Figure 4A

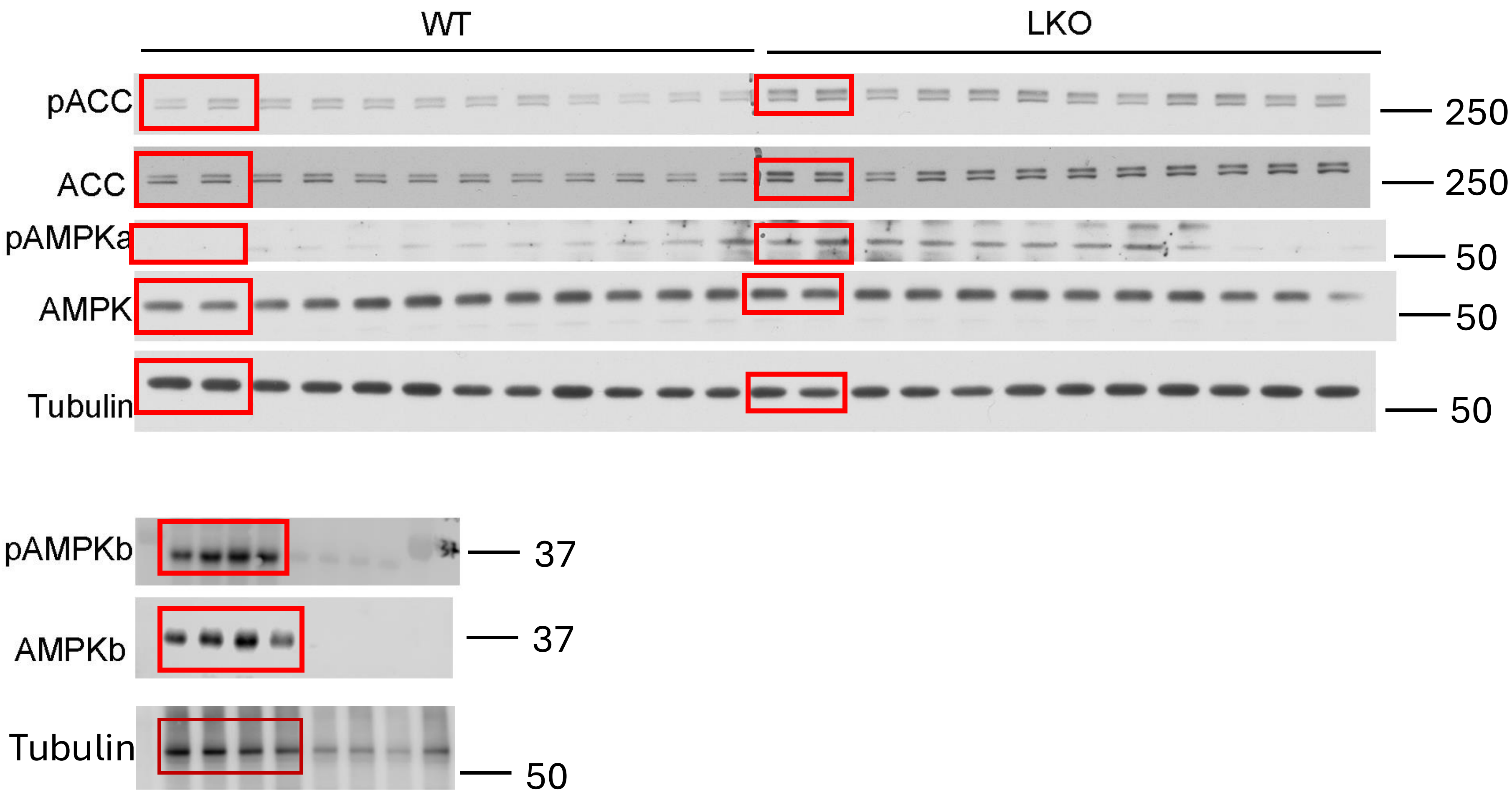
WT
LKO
pACC
ACC
pAMPKa
AMPK
Tubulin
250
250
50
50
50
pAMPKb
AMPKb
Tubulin
37
37
50

Figure 4B

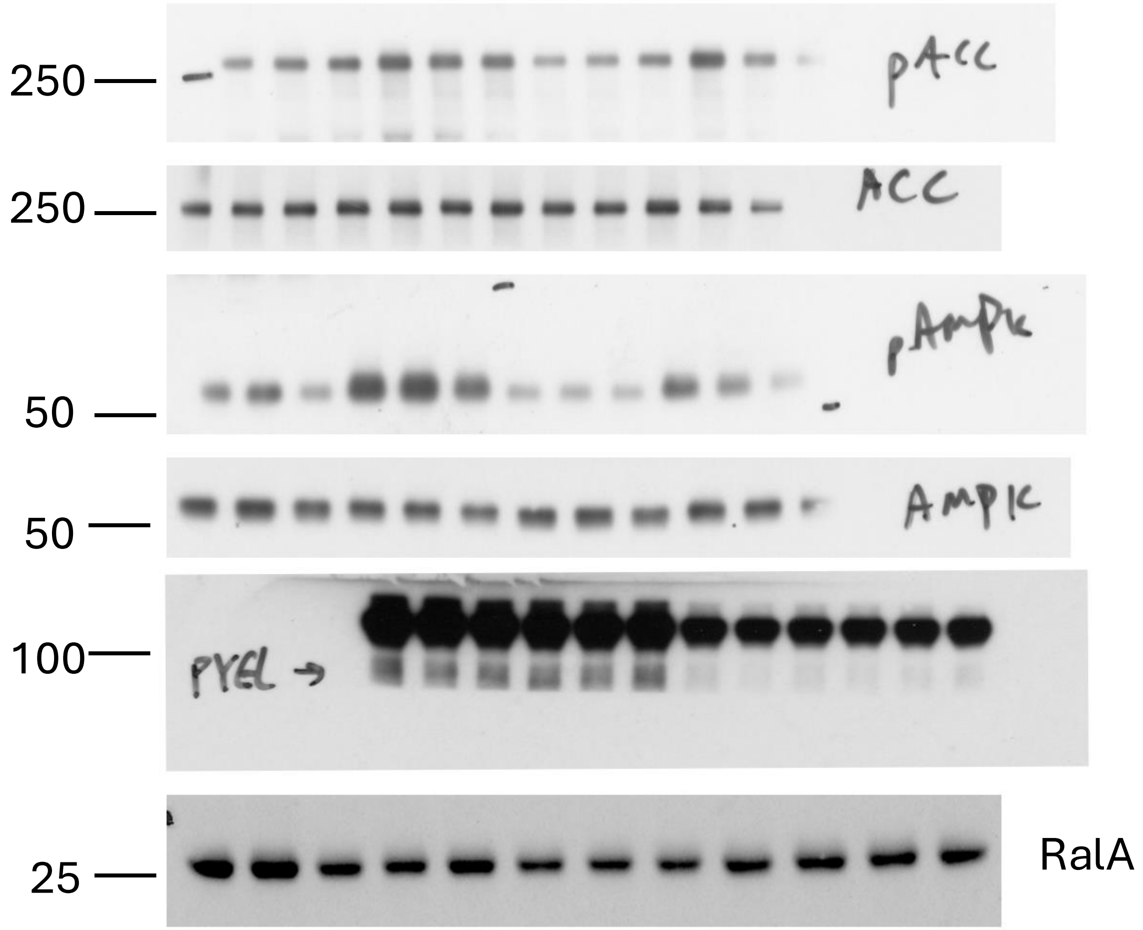

Figure S4C

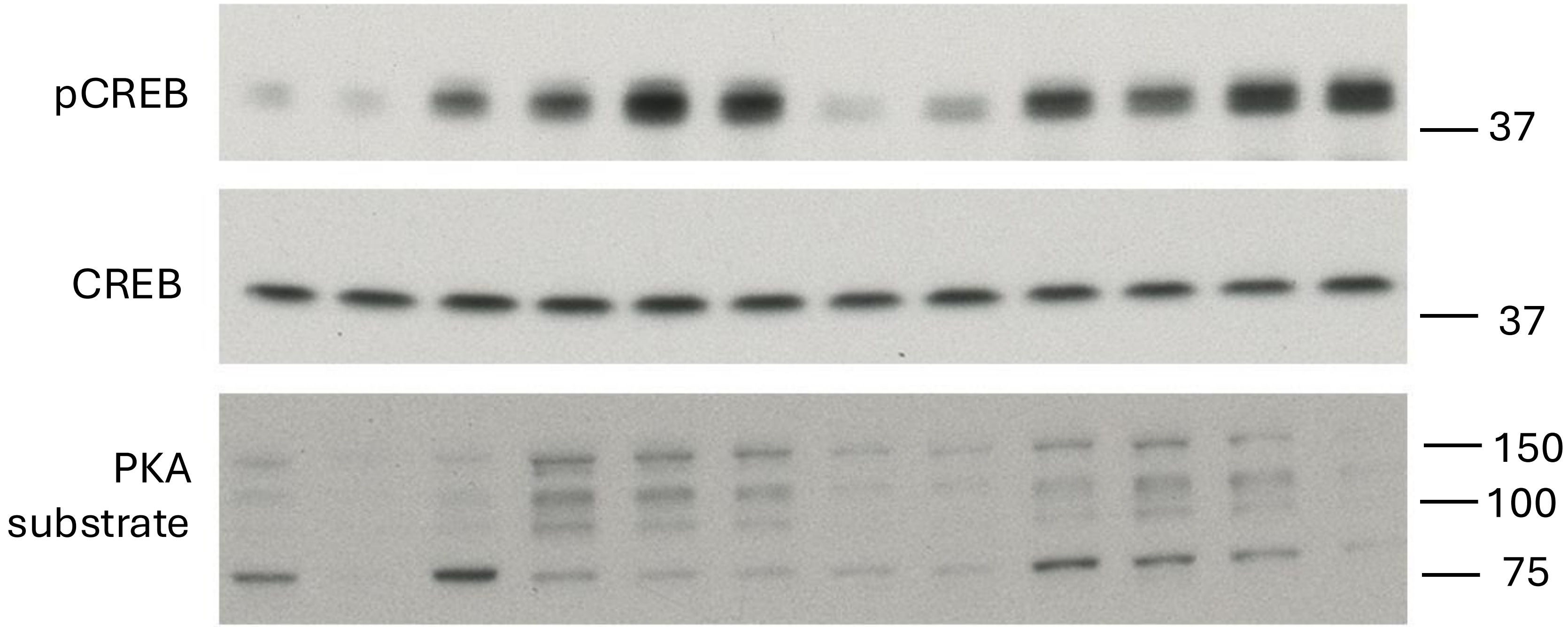

Figure S4D

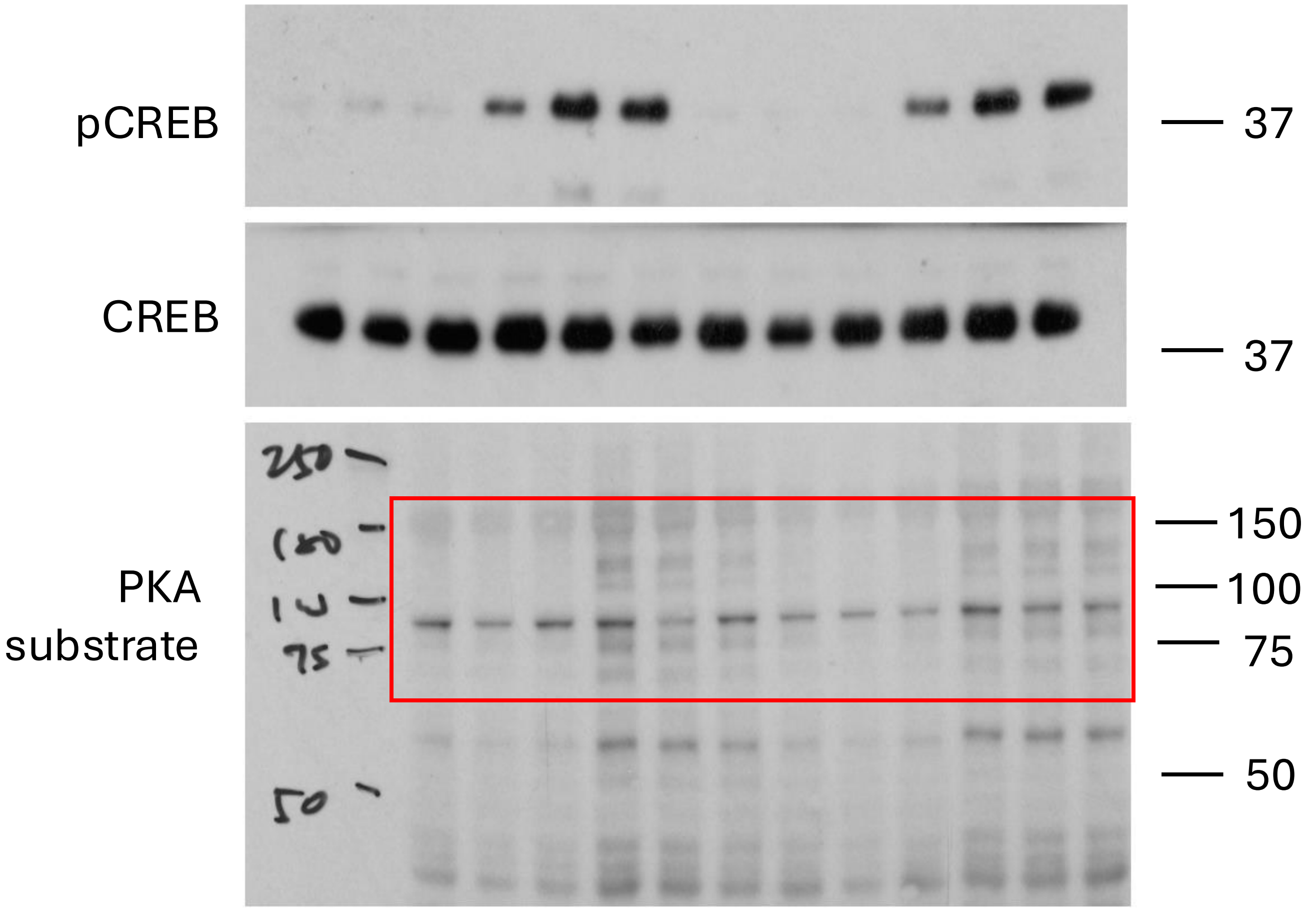

Figure 5A

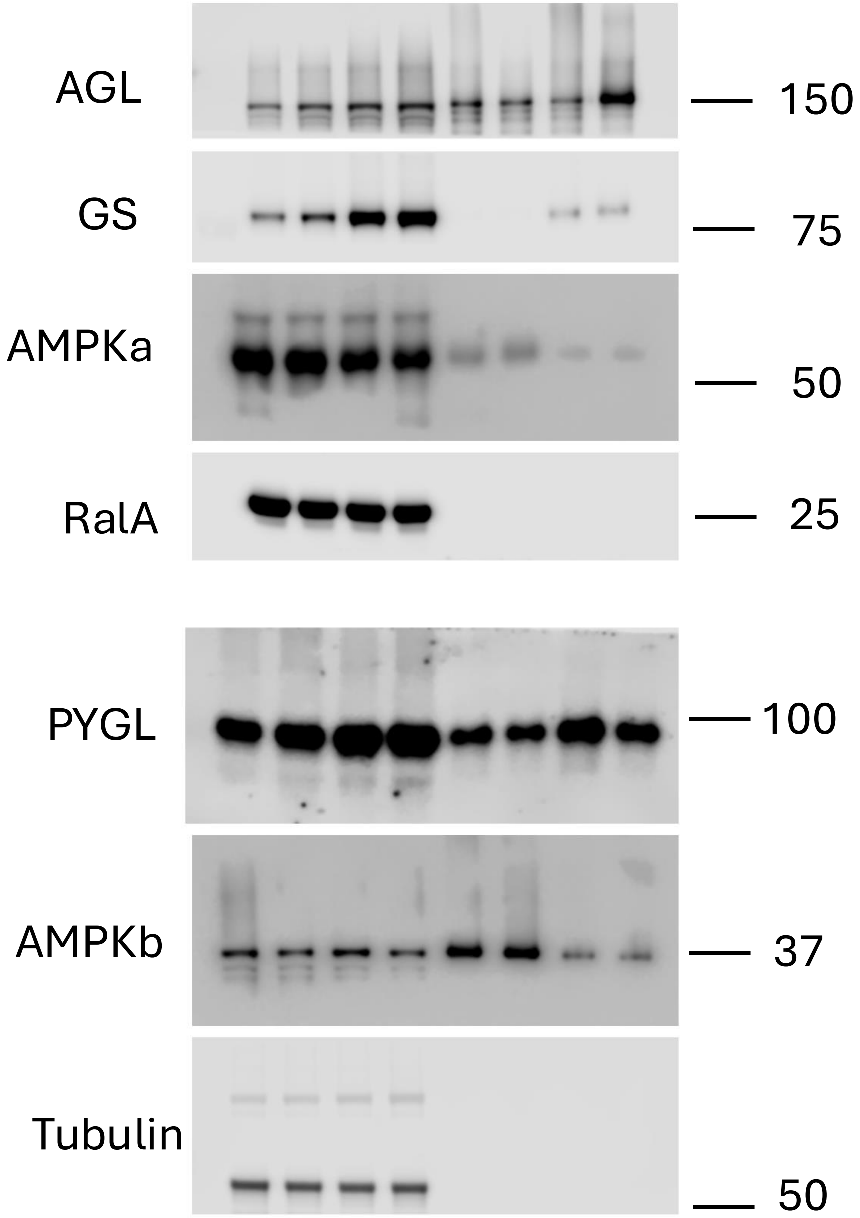

Figure 5B

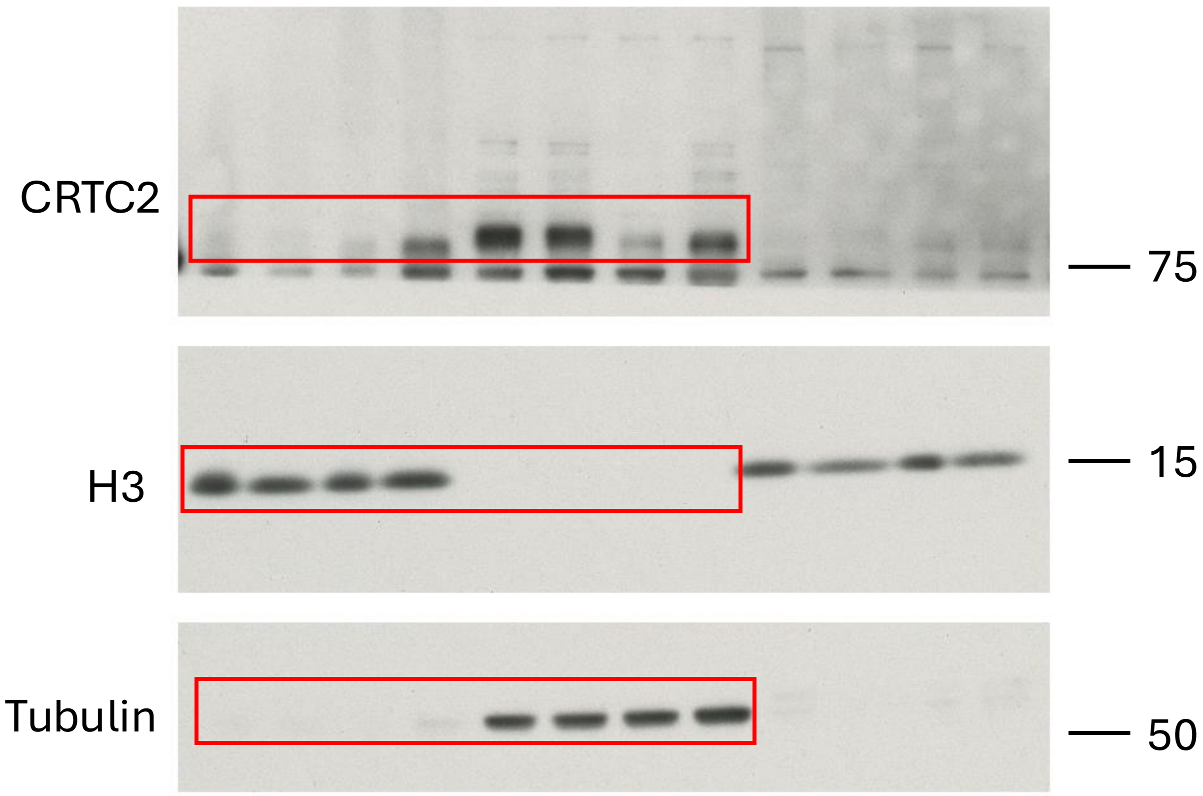

Figure 5F

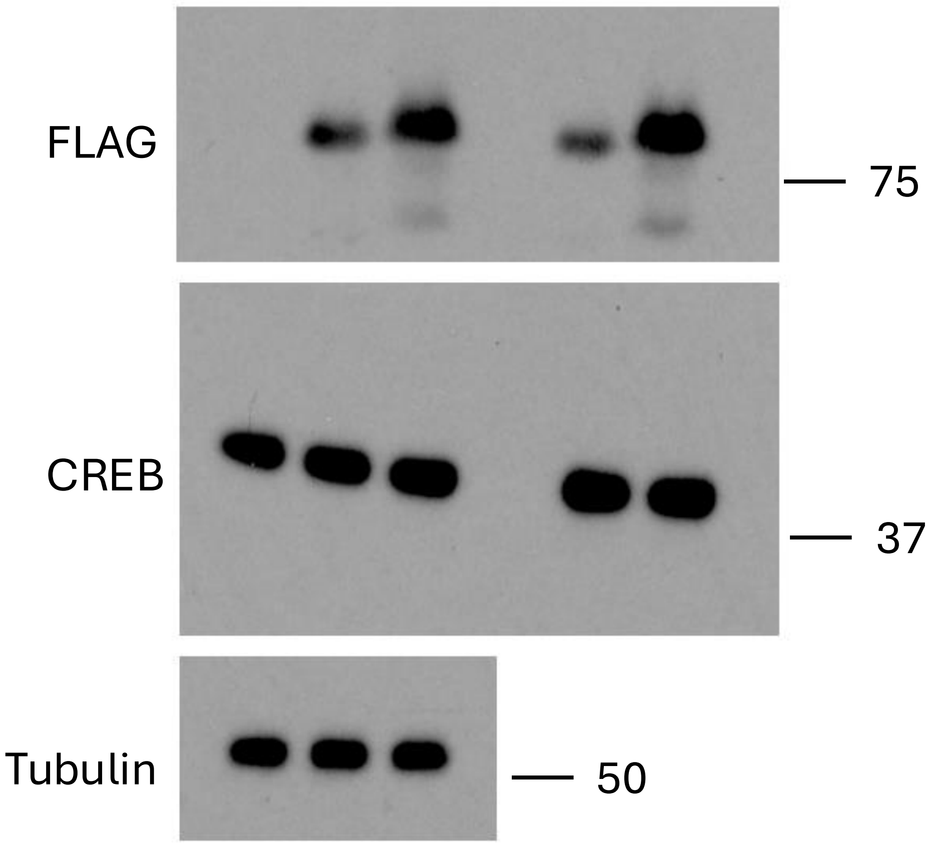

Figure 5G

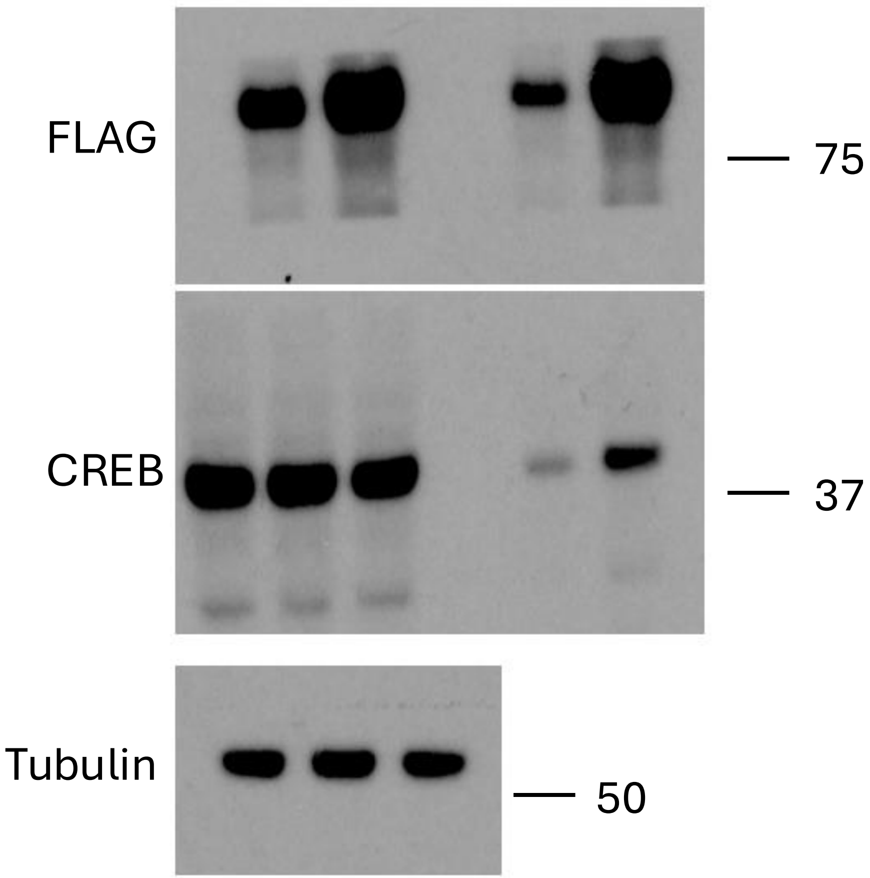

Figure 6A

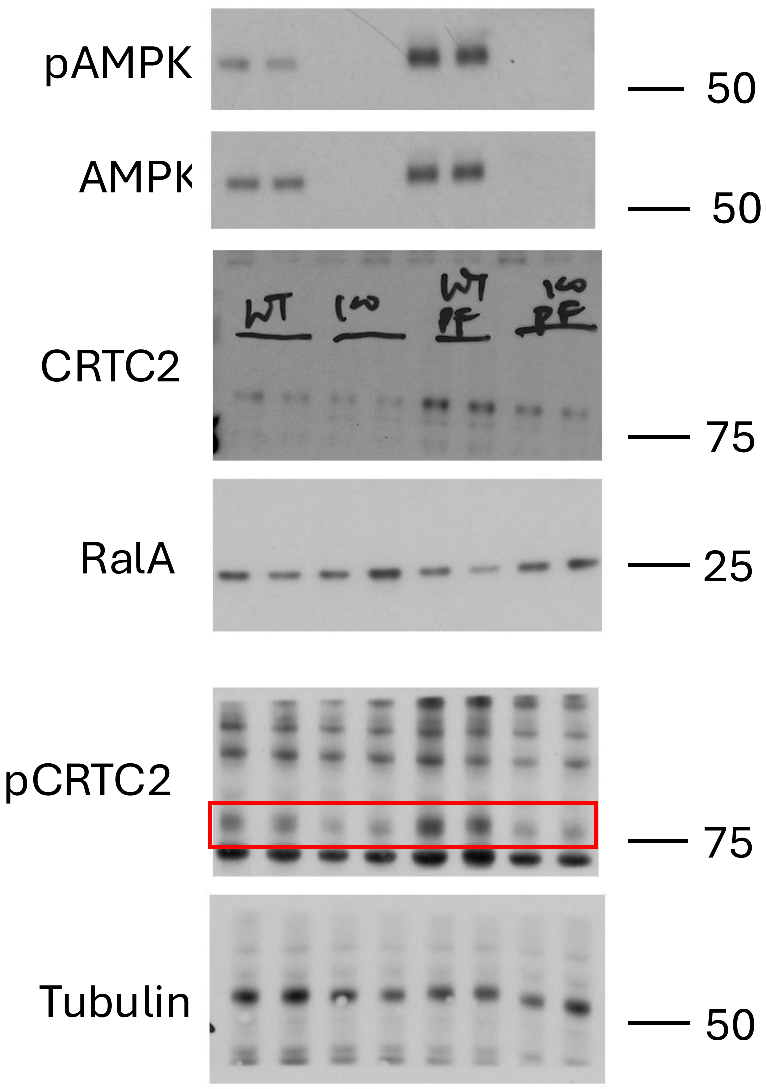
pAMPK
50
AMPK
50
CRTC2
WT
100
WT
PF
100
PF
75
RalA
25
pCRTC2
75
Tubulin
50

Figure 6B

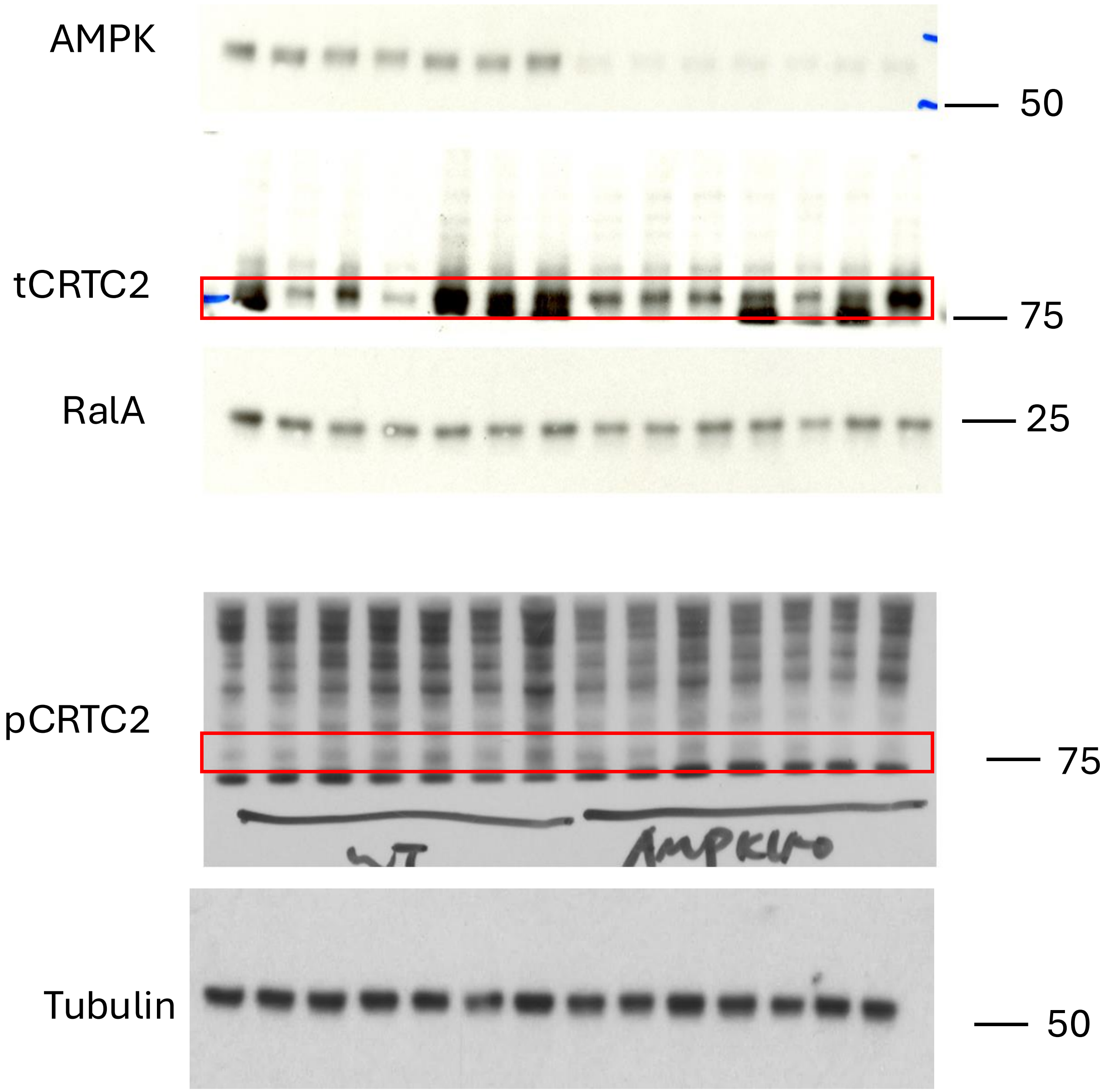

Figure 6C

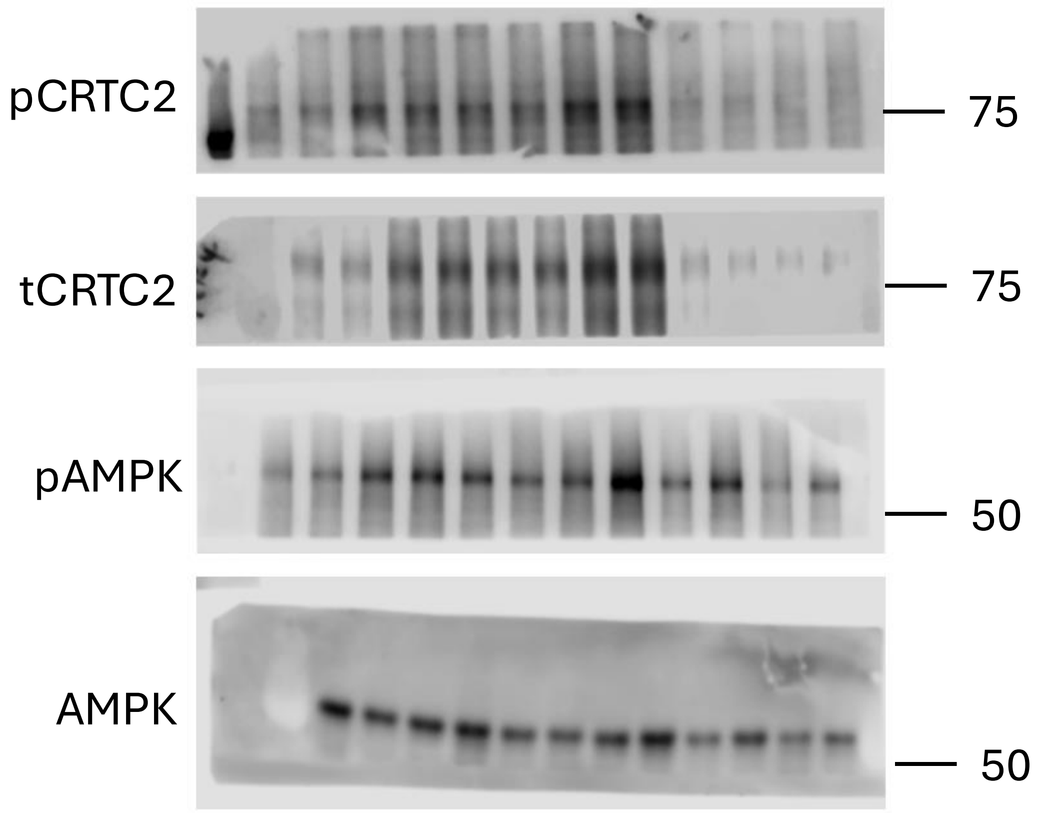

Figure 6D

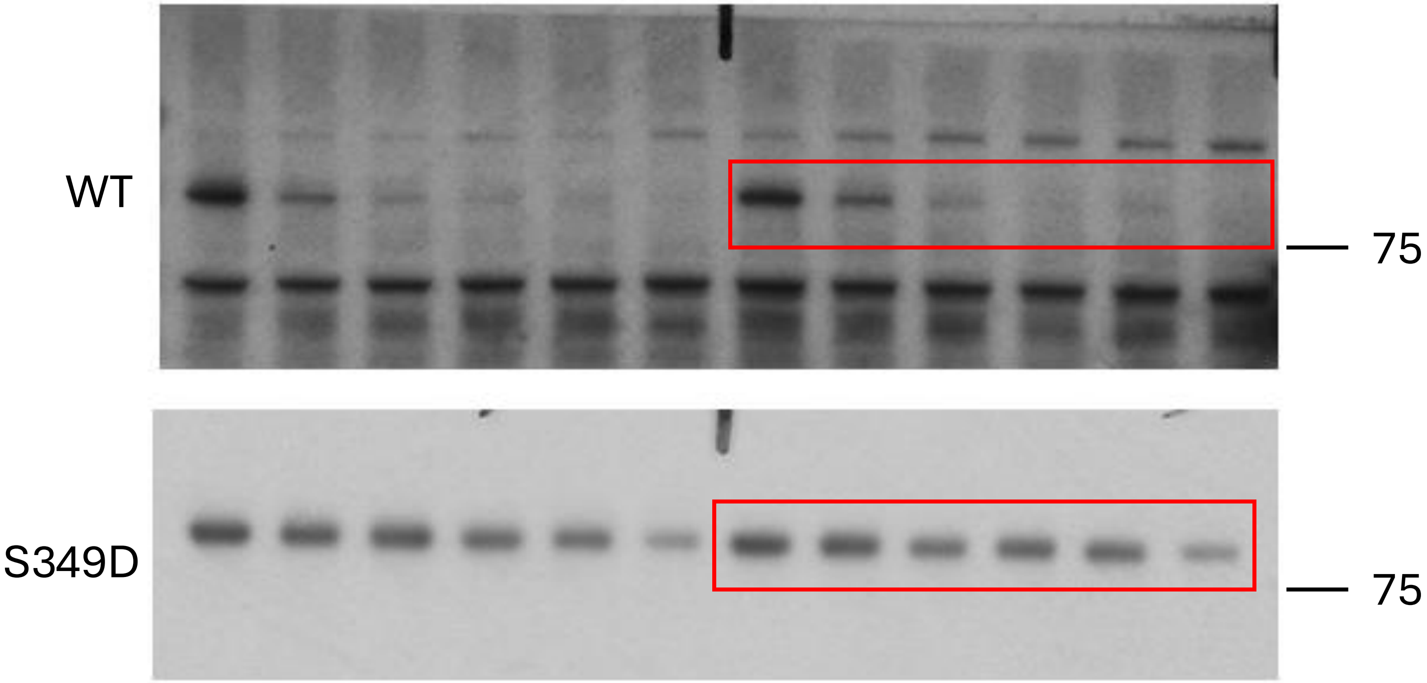

Figure 6E

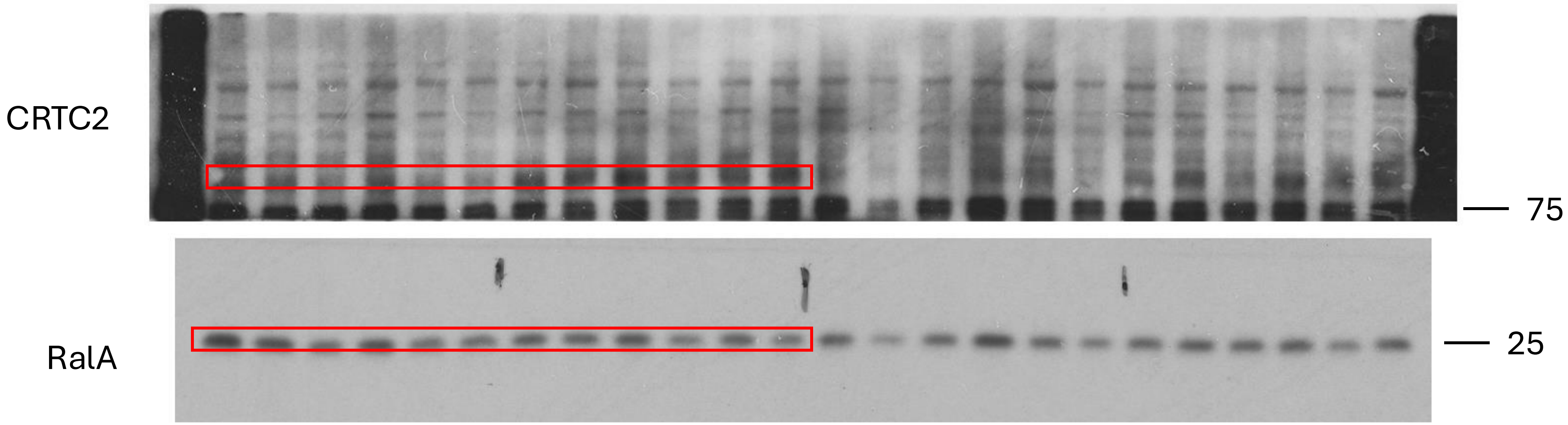

Figure 6F

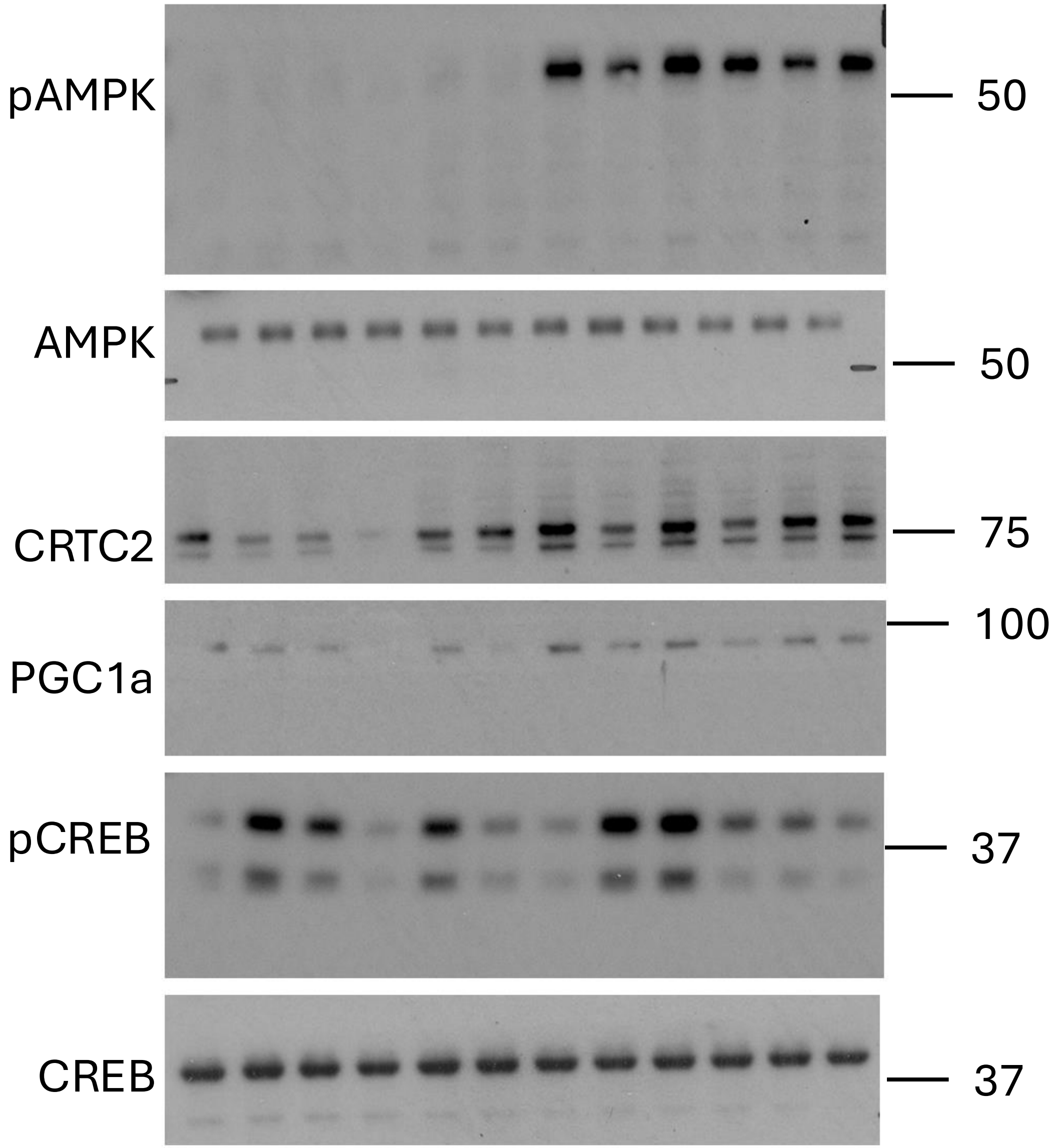

Figure 6G

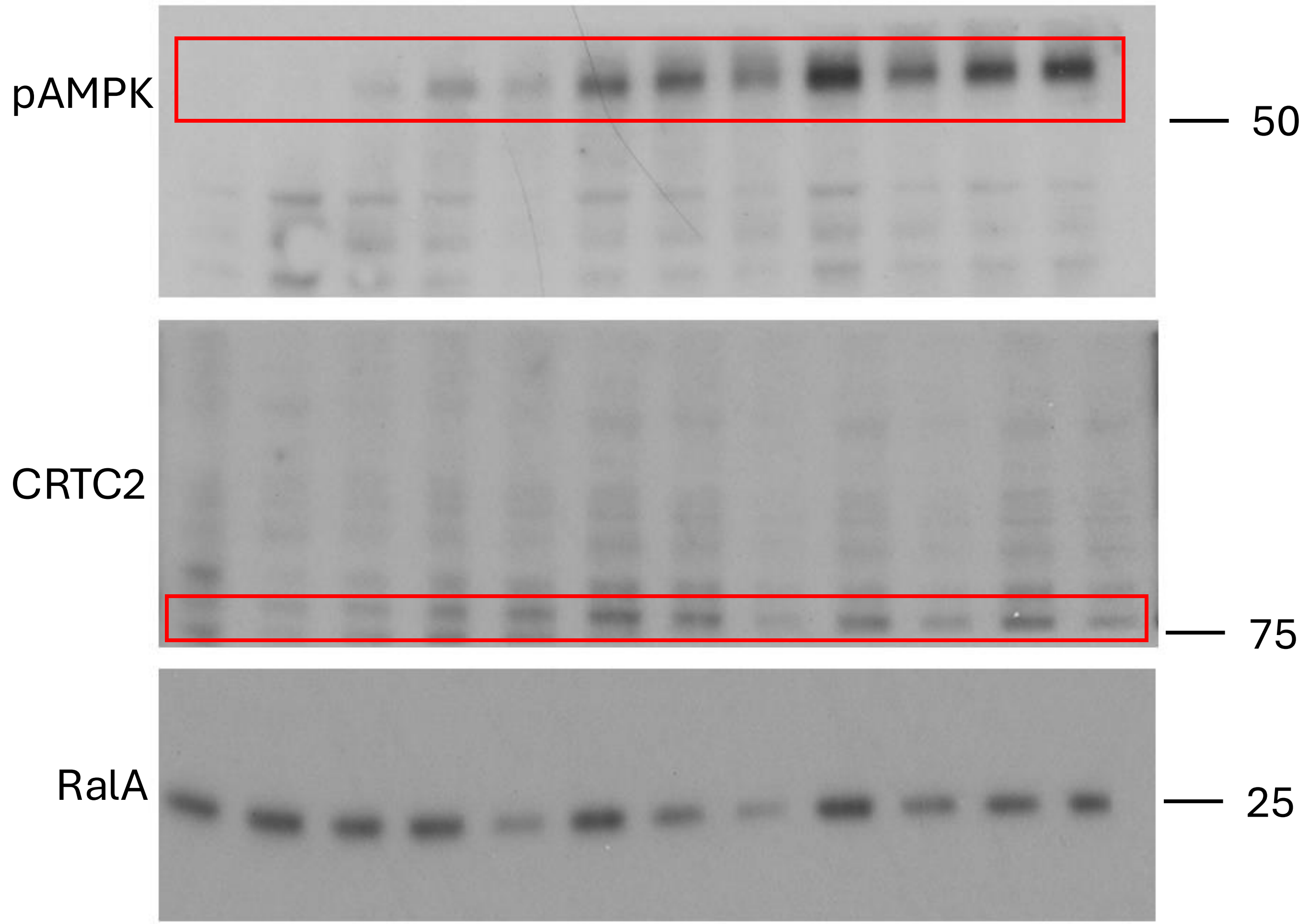

Supplement: Unedited blot and gel images [file jci-135-188363-s341.pdf]
